# YY1-mediated NDUFA9 upregulation promotes NSCLC cell growth through mitochondrial and Akt-mTOR pathway modulation

**DOI:** 10.1038/s41419-026-08562-y

**Published:** 2026-04-21

**Authors:** Yubo Yan, Yuge Zhao, Jun Tang, Xiwen Wang, Jungang Zhao, Yingnan Yang

**Affiliations:** 1 Department of Thoracic Surgery, Harbin Medical University Cancer Hospital, Harbin, China; 2 Department of Thoracic Surgery, Shengjing Hospital to China Medical University, Shenyang, China

**Keywords:** Non-small-cell lung cancer, Oncogenes

## Abstract

NSCLC remains a primary contributor to cancer-related mortality. This study comprehensively elucidates the expression and the functional role of NDUFA9 in NSCLC pathogenesis. Our initial bioinformatic analyses, drawing from TCGA and single-cell RNA sequencing data, revealed a significant upregulation of *NDUFA9* expression within NSCLC tumor tissues and cancer cell populations, correlating with unfavorable clinicopathological indicators, including advanced pathological T stage, male gender, smoking history, and diminished overall survival. Furthermore, NDUFA9 was distinctly enriched in proliferating cancer cells and malignant epithelial cells across diverse metastatic sites. Experimental validation confirmed NDUFA9’s heightened mRNA and protein expression in both locally-treated NSCLC patient tissues and various NSCLC cell types. Functionally, NDUFA9 shRNA or knockout compromised mitochondrial function in NSCLC cells. This impairment was evidenced by a reduced oxygen consumption rate, diminished mitochondrial complex I activity, decreased ATP production, mitochondrial depolarization, reduced mtDNA contents, and an augmented generation of ROS. Concomitantly, NDUFA9 depletion significantly suppressed key malignant phenotypes, including cell proliferation and migration, while inducing apoptosis in NSCLC cells. Conversely, NDUFA9 overexpression in NSCLC cells enhanced mitochondrial function and promoted malignant cellular phenotypes. NDUFA9 was identified as a positive regulator of the Akt-mTOR signaling pathway; its depletion inhibited, and its overexpression enhanced, mTOR kinase activity and the phosphorylation of Akt and S6K in primary NSCLC cells. Bioinformatics predictions, subsequently validated experimentally, established YY1 as a pivotal transcription factor directly binding to and upregulating NDUFA9 expression in NSCLC tissues and cells. Finally, in vivo xenograft studies demonstrated that NDUFA9 silencing suppressed tumor growth, corroborating the in vitro findings by inhibiting mitochondrial function, proliferation, Akt-mTOR activation, and inducing apoptosis within tumor tissues. Thus, NDUFA9 is a crucial regulator of mitochondrial metabolism and malignant progression in NSCLC, driven by YY1-mediated transcriptional control.

## Introduction

Non-small cell lung carcinoma (NSCLC) represents a significant global health burden characterized by substantial morbidity and mortality [1, 2]. exhibiting a complex epidemiology with increasing incidence rates influenced by environmental and genetic factors [2, 3]. Despite advancements in therapeutic modalities encompassing surgical resection, radiation therapy, chemotherapy, targeted therapies, and immunotherapies [4, 5]. NSCLC remains a leading cause of cancer-related deaths worldwide [6, 7]. The classification of NSCLC primarily distinguishes between adenocarcinoma, squamous cell carcinoma, and large cell carcinoma [1, 2].

Currently, targeted therapy for NSCLC is a cornerstone of treatment for patients harboring specific oncogenic driver mutations [2, 3, 8]. Approved targets include EGFR, ALK, ROS1, BRAF (specifically V600E), MET (exon 14 skipping and amplification), RET fusions, NTRK fusions, and KRAS G12C, with corresponding tyrosine kinase inhibitors (TKIs) demonstrating significant improvements in progression-free survival (PFS) and overall survival (OS) in these molecularly defined subsets [2, 4, 5, 9–12]. Furthermore, immune checkpoint inhibitors targeting the PD-1/PD-L1 axis (e.g., pembrolizumab, nivolumab, atezolizumab) and CTLA-4 (ipilimumab) have provided durable responses for a broader patient population, often used as monotherapy or in combination with chemotherapy or targeted agents [13–15]. Angiogenesis inhibitors like bevacizumab and ramucirumab are also being utilized in specific NSCLC settings [16, 17].

Despite these advancements, several limitations persist. Acquired resistance to both targeted therapies and immune checkpoint inhibitors invariably emerges through various mechanisms, including on-target secondary mutations, activation of bypass signaling pathways, and phenotypic transformations [5, 9–12]. Moreover, a significant proportion of NSCLC patients lack identifiable actionable driver mutations, and the efficacy of immunotherapy is limited by primary or acquired resistance mediated by factors within the tumor microenvironment and intrinsic tumor characteristics [5, 9–12]. Consequently, there remains a critical unmet need to identify and validate novel molecular targets, explore combination strategies to overcome resistance, and develop innovative therapeutic approaches for NSCLC patients who do not benefit from current treatment paradigms.

Mitochondria play a pivotal role in the progression of NSCLC by supplying the necessary bioenergetic support and biosynthetic building blocks required for unchecked cellular proliferation and survival within the tumor microenvironment [18, 19]. Mitochondrial proteomic dysregulation constitutes a ubiquitous phenomenon in NSCLC, demonstrably potentiating its aggressive phenotypical manifestation and neoplastic progression [20–25]. NDUFA9 (NADH:ubiquinone oxidoreductase subunit A9) is a nuclear-encoded gene and provides instructions for a protein that serves as an accessory subunit of the mitochondrial respiratory chain complex I [26–29]. The latter is the primary entry point for NADH-derived electrons into the oxidative phosphorylation (OXPHOS) system. While NDUFA9 lacks direct catalytic activity in electron transfer, it plays a crucial role in the proper assembly, structural integrity, and overall stability of the large, multi-subunit complex I holoenzyme embedded within the inner mitochondrial membrane [26–29]. This subunit can facilitate interactions between other complex I subunits and is involved in the intricate process of complex I biogenesis [26–29]. Consequently, genetic defects in NDUFA9 can disrupt complex I function, leading to a variety of mitochondrial disorders characterized by impaired cellular energy production, often manifesting with severe neurological involvement such as Leigh syndrome [27, 29].

Few studies have focused on the role of NDUFA9 in human cancers. Alhawamdeh et al., recently showed that the natural polyphenol Resveratrol significantly reduced the viability of chronic myeloid leukemia (CML) cells and induced apoptosis by upregulating pro-apoptotic genes while causing a slight downregulation of survival-related genes, including NDUFA9 [30]. Zhen et al., reported that the isothiocyanate PEITC induced oxidative cell death and inhibited proliferation in osteosarcoma cells by damaging the mitochondrial respiratory chain complexes, evidenced by the reduction in mitochondrial function and the downregulation of NDUFA9 and other complex components [31]. This study delves into the expression, diverse functions, and underlying mechanisms of NDUFA9 in NSCLC.

## Materials and Methods

### Reagents and chemicals

Essential cell culture reagents, including basal medium, FBS, and antibiotics, were from Hyclone (Logan, UT). Antibodies utilized in this study were from Abcam (Cambridge, UK) and Cell Signaling Tech (Danvers, MA); Compounds including puromycin, polybrene, glucose, and N-acetylcysteine (NAC), were purchased from Sigma-Aldrich (St. Louis, MO). Cigarette smoke exposure (CSE) was prepared from Biyuntian (Wuxi, China). Fluorescence dyes were previously reported and were provided by Dr. Sang [25, 32, 33].

### Cell and tissue acquisition

A549 cells, primary human NSCLC cells (pNSCLC1/2/3), and primary lung epithelial cells (pEpi1/2) were acquired via established protocols and were generated from Dr. Sang [25, 32, 33]. All cells were screened for mycoplasma, authenticated by STR profiling, and morphologically assessed. NSCLC tumors and adjacent normal lung tissues were obtained from consenting primary lung cancer patients at the authors' institutions, with all human protocols ethically sanctioned and approved by the Ethics Board of Harbin Medical University Cancer Hospital (HYDZLR-2023062), adhering to the Helsinki Declaration.

### Quantitative real-time PCR (qRT-PCR)

Total RNA was isolated from cellular or tissue lysates using TRIzol reagent and reverse transcribed into cDNA. Amplification followed standardized methodologies [23]. with *GAPDH* as the internal normalization reference. Quantitative data analysis was performed using previously validated protocols [23]. Primers were verified and provided by Genechem (Shanghai, China).

### Western blot

Cellular and tissue lysates were separated by SDS-PAGE (7.5% to 12.5% acrylamide gels) and transferred to PVDF membranes. Membranes were blocked with 5% non-fat milk in PBST for 45 min, then incubated overnight at 4 °C with primary antibody solutions. After washing, membranes were incubated with HRP-conjugated secondary antibody solution for 55 min. Protein bands were visualized via enhanced chemiluminescence, and densitometric quantification was performed using ImageJ. Uncropped blot images are in Supplementary Fig. S1.

### Gene silencing and overexpression

Lentiviral constructs (using the GV369 construct from Genechem) were engineered for gene silencing and overexpression of human *NDUFA9* or *YY1* (*Yin Yang 1*). For knockdown, two distinct shRNAs targeting NDUFA9 (shNDUFA9-sh1 and shNDUFA9-sh2) and two distinct shRNAs targeting YY1 (shYY1-sh1 and shYY1-sh2) were employed. Overexpression studies utilized constructs encoding the NDUFA9 and YY1 cDNA sequences. These constructs were co-transfected with lentivirus envelope constructs (Genechem) into HEK-293 cells using Lipofectamine 2000 to produce lentiviral particles. The resulting particles (at MOI 10) were transduced to NSCLC or lung epithelial cells for 40 h. Cells were subsequently maintained in complete medium supplemented with polybrene, and stable cells were established through five-six passages of puromycin selection. The efficacy of silencing or overexpression of targeted genes was validated at both mRNA and protein levels.

### CRISPR/Cas9-mediated gene knockout (KO)

Precise genetic ablation of NDUFA9 was achieved via CRISPR/Cas9-mediated knockout (KO). NSCLC cells were initially transduced with Cas9-expressing lentiviral particles (provided by Dr. Cao [34, 35]) to ensure constitutive Cas9 nuclease expression, followed by the establishment of stable Cas9-expressing cells through puromycin selection. Two distinct sgRNAs specifically targeting human NDUFA9 (koNDUFA9-sg1 or koNDUFA9-sg2) were cloned into a lenti-CRISPR/Cas9-KO-puro construct (Genechem, Shanghai, China). Lentiviral particles harboring these constructs then transduced the previously established stable Cas9-expressing NSCLC cells. Stable “koNDUFA9” cells were selected using puromycin, and individual cell clones were established via single-cell cloning. “sgC” control cells, carrying a non-targeting sgRNA, were previously characterized and were from Dr. Sang [25, 32, 33].

### Quantification of the GSH/GSSG ratio

The redox status was assessed through quantification of the GSH/GSSG ratio, employing a specialized kit from Thermo-Fisher Scientific (Suzhou, China). Prepared lysates were incubated with DTNB, glutathione reductase, and NADPH. Spectrophotometric measurements of absorbance at 425 nm were conducted over an interval of five minutes. Concentrations were precisely determined via a standard curve established using GSH and GSSG reference standards.

### Thiobarbituric acid reactive substances (TBAR) assay

Levels of lipid peroxidation were evaluated utilizing a TBARS assay kit from Thermo-Fisher Scientific (Suzhou, China). Lysates derived from tissues or cells were combined with thiobarbituric acid (TBA) to generate the TBAR adduct. Subsequent to cooling and centrifugation for precipitate removal, optical density was quantified at 525 nm via spectrophotometry.

### Mitochondrial complex I activity

Mitochondrial complex I enzymatic kinetics were precisely determined using a commercial kit from Sigma. This method involved spectrophotometrically measuring the NADH to NAD+ conversion catalyzed by complex I. Activity was quantified by the attenuation of absorbance at 435 nm.

### ATP level quantification

Intracellular and tissue ATP concentrations were quantified using a commercial colorimetric kit from Sigma, strictly following the manufacturer’s protocol. Each assessment utilized 25 µL of cellular or tissue homogenate (25 µg total protein).

### mtDNA measurement

The mitochondrial DNA (mtDNA) abundance was determined using either comprehensive cellular lysates or pristine tissue specimens. We isolated total DNA through the well-established phenol-chloroform extraction method. Subsequently, quantitative Polymerase Chain Reaction (qPCR) was executed utilizing oligonucleotide primers specifically designed for the mitochondrial gene MT-ND1 (*NADH dehydrogenase subunit 1*) and the nuclear endogenous control gene *GAPDH*. The relative mtDNA copy count was computed via the ΔΔCt analytical approach, with MT-ND1 cycle threshold (Ct) values standardized against GAPDH Ct values. All PCR amplification steps adhered to conventional thermocycling protocols, and a post-amplification melt curve assessment was performed to corroborate the specificity of the primers.

### Akt1 mutation

We introduced lentiviral particles containing the constitutively-active Akt1 mutant (S473D, caAkt1), provided by Dr. Cao’s group [36–38]. into cultured NSCLC cells. Following puromycin selection, we established stable NSCLC cells expressing caAkt1.

### mTOR kinase activity assay

NSCLC cells, with specific NDUFS9 genetic modifications, were engineered to stably express FLAG-tagged mTOR (from Dr. Jiang [21]. Cells were lysed in ice-cold CHAPS buffer, and clarified lysates underwent immunoprecipitation with anti-FLAG agarose beads for 90 min at 4 °C. Beads were pelleted (500 × *g*, 45 s), washed three times with ice-cold buffer [39]. and re-suspended in mTOR kinase assay buffer containing 750 µM ATP to initiate GST-tagged p70S6K1 phosphorylation. Reactions ceased after 12 min [39]. and we quantified phospho-p70S6K (Thr389) levels using an anti-phospho-p70S6K antibody (Cell Signaling Tech), HRP-conjugated secondary antibody, and TMB substrate. Absorbance (450 nm) was measured, and kinase activity was normalized to control samples.

### Cellular fluorescence staining

Cells were inoculated into 24-well plates at 1.5–2.5 × 10^4^ cells per well in 550 µL of basal medium and incubated for a pre-specified duration [25]. Following incubation, cells underwent chemical fixation with 4% paraformaldehyde and extensive washing with cold PBS. Cells were then incubated with specific fluorochromes, followed by additional PBS washes. Visualization was performed using a Leica fluorescence microscope, with fluorescence emission intensity quantitatively measured via a Hitachi F-7000 spectrophotometer.

### Oxygen consumption rate (OCR)

OCR was ascertained using an Agilent Seahorse XF24 Extracellular Flux Analyzer, adhering rigorously to previously listed protocols [40]. Cellular respiration was comprehensively elucidated by sequentially perfusing cells with specific metabolic modulators: 1 µM oligomycin, followed by 0.5 µM FCCP (carbonyl cyanide-p-trifluoromethoxyphenylhydrazone), and concluding with a conjoint application of 0.5 µM antimycin A and rotenone. This systematic pharmacological perturbation facilitated the comprehensive quantification of basal, ATP-linked, maximal, and non-mitochondrial OCR components. All derived OCR values were normalized to the intracellular protein content.

### Chromatin Immunoprecipitation (ChIP)

ChIP was performed to examine the interaction between YY1 and the *NDUFA9* promoter. The experimental protocol followed established methodologies [34, 41]. Briefly, cell and tissue lysates were homogenized, and genomic DNA was fragmented. These diluted lysates then underwent immunoprecipitation using an anti-YY1 antibody to enrich for YY1-bound DNA fragments. Finally, quantitative PCR (qPCR) was used to assess the association between YY1 and the predictive *NDUFA9* promoter DNA sequence, with results normalized to control values.

### Additional assays

A series of functional cellular assays was executed as detailed in early studies [25, 32, 33]. Genetically modified NSCLC cells or lung epithelial cells were seeded at a density of 3000 cells per well in 96-well plates and subsequently incubated for 96 h. Next, CCK-8 mixture was introduced, followed by an additional 90 min incubation, with absorbance gauged at 450 nm using a microplate reader. For colony formation assays, cells were seeded at a density of 3000 cells per well in 6-well plates. Cells were cultured in complete growth medium and incubated for 10 days. Medium was refreshed every 3 days. Subsequently, colonies were fixed, stained, and then washed, and were counted manually. Cell death was determined via Trypan blue staining using an automatic cell counter, subsequent to designated treatments. The cytosol cytochrome C assay in prepared lysates was assessed utilizing a colorimetric assay kit (Cell Signaling Tech, Danvers, MA), strictly observing the manufacturer’s stipulations. For Transwell assays, genetically modified NSCLC cells were suspended at 1.2×10^4^ cells/well in serum-free medium and added to Transwell chambers. After 24 h, migrated cells on the lower surface were fixed, stained, and photographed.

### Animal xenograft studies

The xenograft experiments were undertaken using 6-week-old nude mice (18.3-18.9 g), with an equitable distribution of male and female subjects. These mice were housed at the Animal Facility at Soochow University (Suzhou, China). Exactly seven million pNSCLC-1 cells per mouse were subcutaneously (*s.c*.) injected into the flanks. Tumor dimensions were measured, and volumes were computed using a previously delineated formula [25, 33]. For immunohistochemistry (IHC), we first prepared paraffin-embedded xenograft tissue sections by baking, deparaffinization, rehydration, and extensive PBST washes. To mitigate non-specific binding, sections were blocked with 5–6% serum. Endogenous peroxidase activity was then inactivated with hydrogen peroxide. Primary antibody incubation proceeded for 6–7 h at room temperature, followed by 1.5 h with biotin-conjugated secondary IgG antibodies. After thorough washing, antigen-antibody complexes were visualized using diaminobenzidine (DAB) chromogen. All animal experimental protocols received ethical imprimatur from the Institutional Animal Care and Use Committee (IACUC) and the Ethics Board of Harbin Medical University Cancer Hospital (HYDZLR-2023062).

### Statistical analysis

For in vitro studies, a blinded approach was used for group allocation, and experiments were repeated across five distinct biological replicates. Normally distributed data are presented as mean ± standard deviation (SD). SPSS version 23.0 (SPSS Co., Chicago, IL) was utilized for all statistical analyses. Unless otherwise mentioned, we used the unpaired Student’s t-test for comparisons between two groups. For comparisons involving more than two groups, one-way ANOVA was employed, followed by Scheffe’s and Tukey post-hoc tests. A *P*-value less than 0.05 indicated statistical significance.

## Results

### NDUFA9 expression is upregulated in NSCLC and correlates with clinicopathological features and poor prognosis

**Fig. 1:**
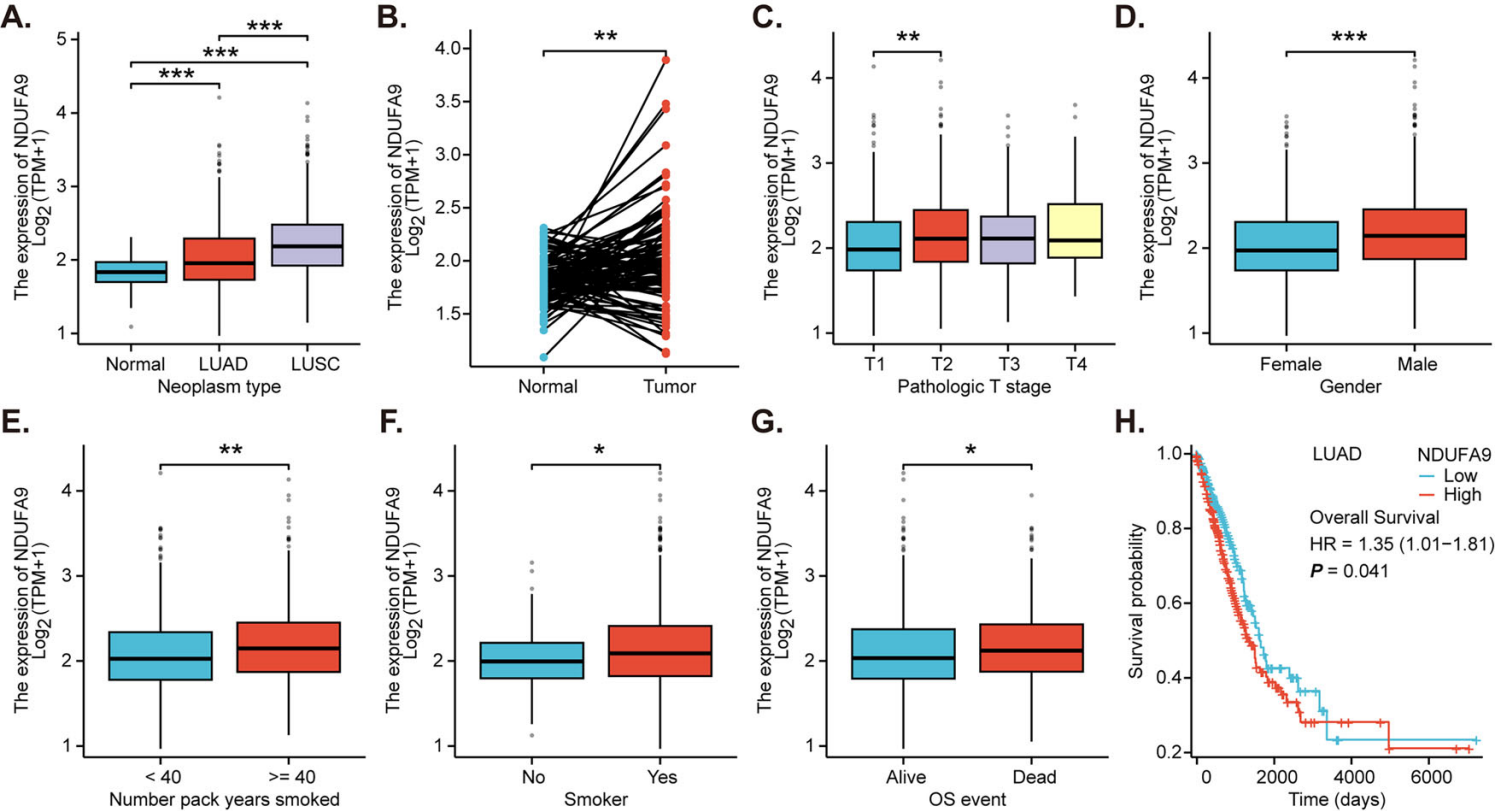
NDUFA9 expression is upregulated in NSCLC and correlates with clinicopathological features and poor prognosis. Box plots illustrating *NDUFA9* mRNA expression levels in NSCLC tumor tissues (LUAD and LUSC) compared to normal lung tissues, and between LUAD and LUSC subtypes using The Cancer Genome Atlas (TCGA) data (**A**). Paired analysis of *NDUFA9* mRNA expression in NSCLC tumor tissues and their corresponding adjacent normal tissues from TCGA (**B**). Box plots showing the relationship between *NDUFA9* mRNA expression and pathological T stage in NSCLC (**C**). Box plots comparing *NDUFA9* mRNA expression levels between male and female NSCLC patients (**D**). Box plots depicting *NDUFA9* mRNA expression in NSCLC patients categorized by smoking status (**E**, **F**). Box plots comparing *NDUFA9* mRNA expression levels in NSCLC patients grouped by overall survival status (Alive vs. Dead) (**G**). Kaplan-Meier survival curve illustrating the overall survival probability of LUAD patients stratified by high and low *NDUFA9* expression levels (**H**). The hazard ratio (HR) and 95% confidence interval (CI) were calculated using Cox proportional hazards regression analysis, and the *P*-value was determined by the log-rank test.

To elucidate the clinical significance of *NDUFA9* expression in NSCLC, we analyzed transcriptomic data from The Cancer Genome Atlas (TCGA), focusing on the lung adenocarcinoma (LUAD) and lung squamous cell carcinoma (LUSC) subtypes. Our analysis revealed a statistically significant upregulation of *NDUFA9* in NSCLC tumor tissues compared to normal lung tissues (Fig. 1A). *NDUFA9* expression levels were significantly elevated in LUSC samples relative to LUAD samples (Fig. 1A). The paired tissue analyses demonstrated a significant increase in *NDUFA9* expression within NSCLC tumor tissues compared to their corresponding adjacent normal tissues (Fig. 1B). We also explored the relationship between *NDUFA9* expression and clinicopathological features. *NDUFA9* expression showed a trend of increasing with advancing pathological T stage, with a statistically significant increase observed between T1 and T2 stages (Fig. 1C). In addition, male patients exhibited significantly higher *NDUFA9* expression compared to female patients (Fig. 1D), and smokers (Fig. 1E, F) showing significantly elevated *NDUFA9* expression compared to non-smokers. The expression levels of *NDUFA9* were compared between patients with different overall survival (OS) events. As depicted in Fig. 1G, its expression was significantly lower in the “Alive” group compared to the “Dead” group. This finding suggests a potential association between elevated *NDUFA9* expression and poor overall survival outcomes. Indeed, the Kaplan-Meier survival analysis indicated that high *NDUFA9* expression was significantly associated with poorer overall survival in the LUAD cohort (Fig. 1H). Thus, TCGA data demonstrate that *NDUFA9* is overexpressed in NSCLC compared to normal tissues, correlating with advanced T stage, male gender, and smoking history. Moreover, higher *NDUFA9* expression in NSCLC is associated with worse overall survival.

### Single-cell sequencing shows *NDUFA9* overexpression in the cancer cell population of NSCLC

**Fig. 2:**
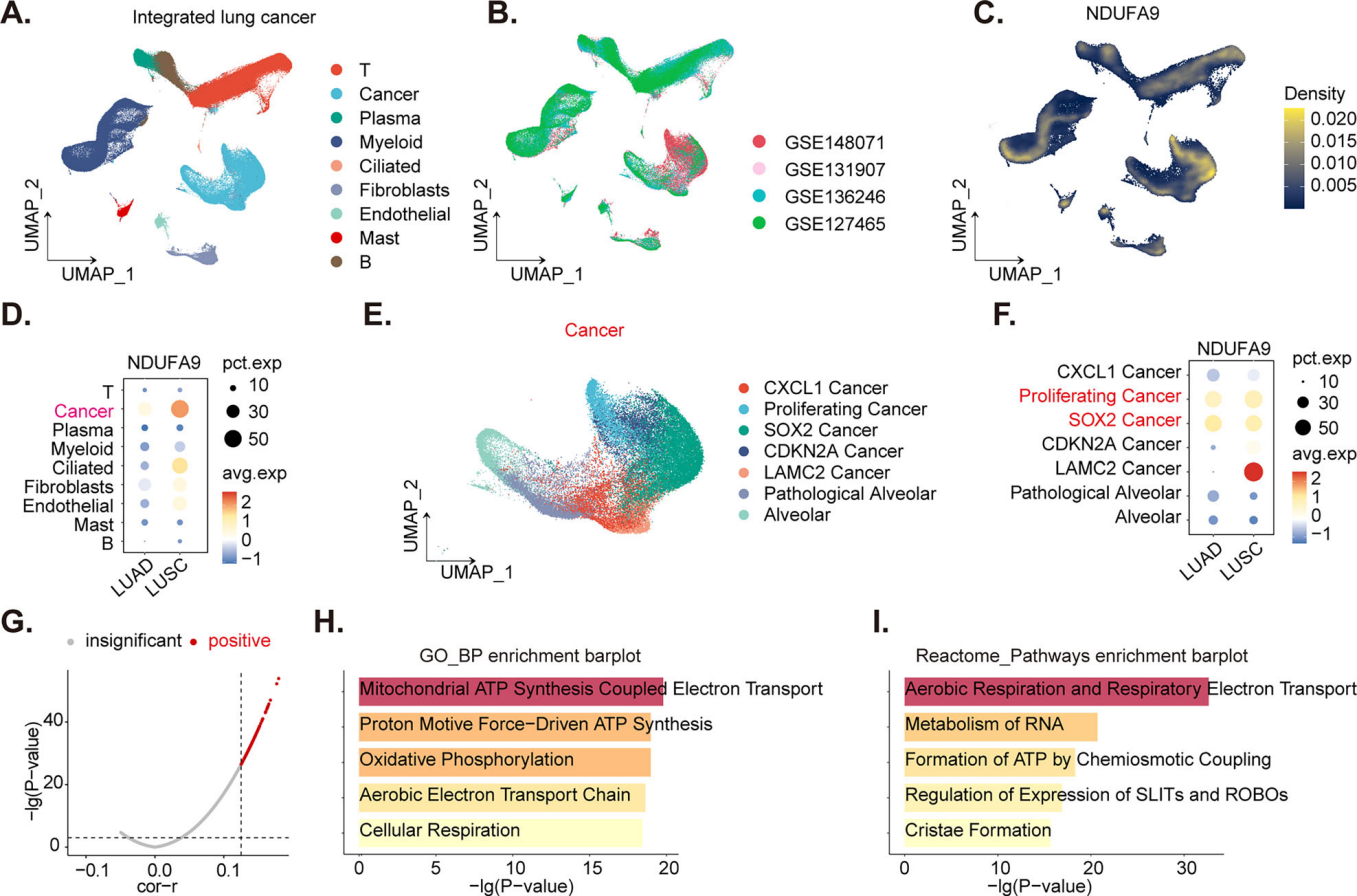
Single-cell sequencing shows *NDUFA9* overexpression in the cancer cell population of NSCLC. Distinct cell populations within the integrated NSCLC single-cell RNA sequencing dataset are visualized in the Uniform Manifold Approximation and Projection (UMAP) plot (**A**). The contribution of different datasets to the integrated analysis is illustrated in the UMAP plot (**B**). The expression of *NDUFA9* across all cells in the integrated NSCLC dataset is shown in the density plot (**C**). A dot plot comparing *NDUFA9* expression (average expression and percentage of expressing cells) across different cell types in LUAD and LUSC is presented (**D**). The UMAP plot of the isolated and sub-clustered cancer cell population, colored by subclusters, is displayed (**E**). A dot plot showing *NDUFA9* expression across the identified cancer cell subclusters, highlighting enrichment in proliferating and SOX2-positive cancer cell subpopulations, is provided (**F**). The volcano plot displaying the correlation of gene expression with *NDUFA9* within tumor cell subclusters, with the top 100 positively correlated genes highlighted, is shown (**G**). The top Gene Ontology (GO) biological processes enriched among the genes positively correlated with *NDUFA9* are depicted in the bar plot (**H**). The top Reactome pathways enriched among the genes positively correlated with *NDUFA9* are illustrated in the bar plot (**I**).

To investigate *NDUFA9* expression patterns and associated pathways in NSCLC, we analyzed an integrated single-cell RNA sequencing dataset, with cell type annotations provided by the original authors [42]. Dimensionality reduction visualized the distinct cell populations within the integrated NSCLC dataset (Fig. 2A), and the contribution of different datasets to the integrated analysis (Fig. 2B). Expression density mapping (Fig. 2C) revealed that *NDUFA9* is predominantly expressed in cancer cells. Dot plot analysis (Fig. 2D) further demonstrated elevated *NDUFA9* expression in the cancer cell population of NSCLC, with a comparatively higher expression level observed in LUSC relative to LUAD (Fig. 2D). Subsequent isolation and sub-clustering of the cancer cell population (Fig. 2E, F) identified *NDUFA9* as highly expressed within this compartment, particularly enriched in the proliferating cancer cell subpopulation and SOX2 cancer cell subpopulation (Fig. 2E, F). Correlation analysis of *NDUFA9* expression within the tumor cell subclusters identified the top 100 positively correlated genes (Fig. 2G). Functional enrichment analysis of these co-expressed genes using Gene Ontology (GO) terms (Fig. 2H) and Reactome pathways (Fig. 2I) revealed significant enrichment in processes related to cellular respiration, mitochondrial ATP synthesis coupled electron transport, oxidative phosphorylation (OXPHOS), and mitochondrial cristae formation (Fig. 2H, I), suggesting a potential critical role for *NDUFA9* in mitochondrial metabolism of NSCLC. These findings underscore the prominent expression of *NDUFA9* in cancer cells of NSCLC.

### *NDUFA9* upregulation in malignant epithelial cells across LUAD metastatic sites

**Fig. 3:**
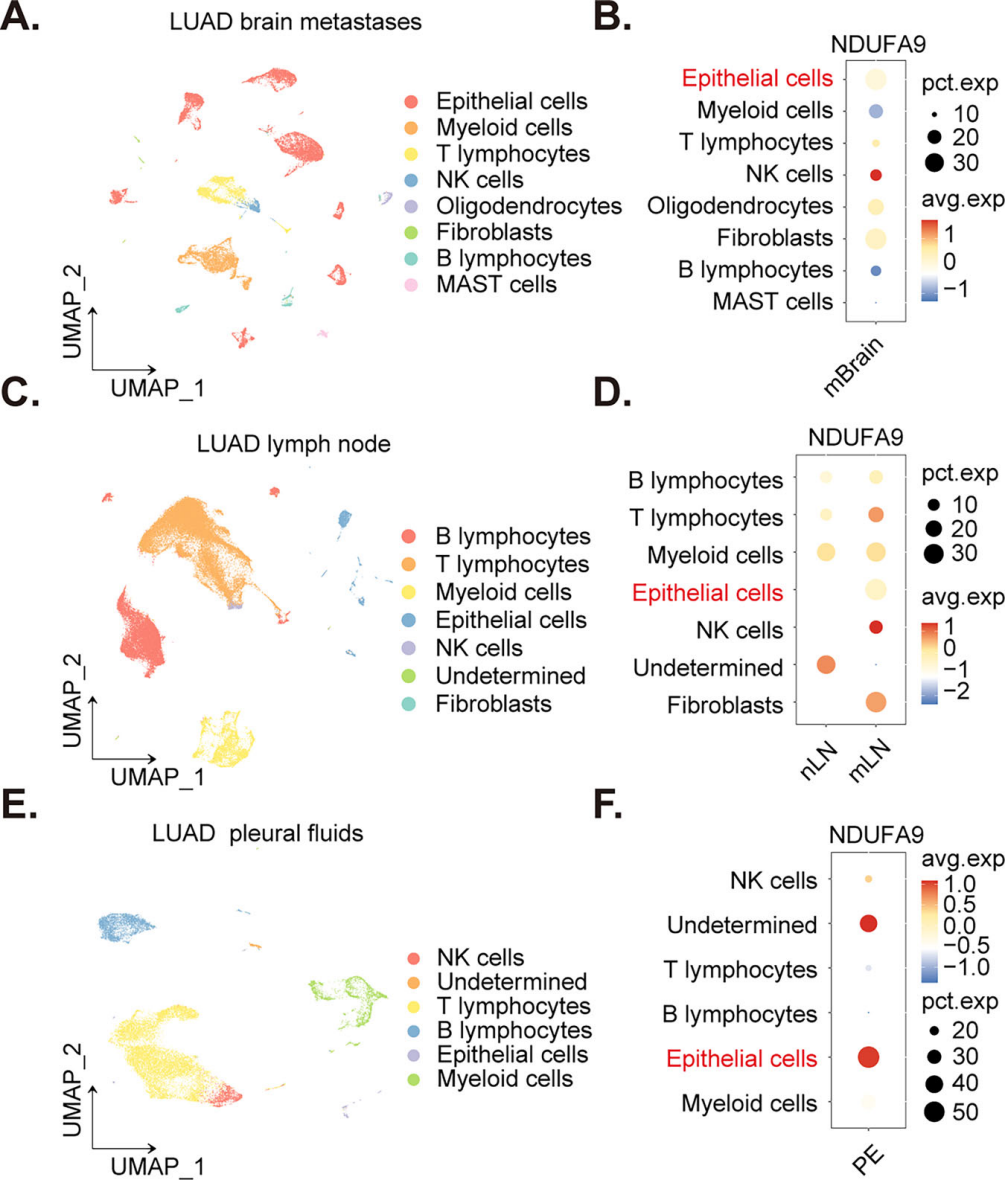
*NDUFA9* upregulation in malignant epithelial cells across LUAD metastatic sites. Uniform Manifold Approximation and Projection (UMAP) plots visualizing single-cell transcriptomic data from GSE131907 of LUAD brain metastases (**A**) lymph node metastases (**C**) and pleural effusions (PE, **E**). Each dot represents a single cell, colored by the indicated cell types. Dot plots (**B**, **D**, and **F**) show the expression of *NDUFA9* across different cell types within each metastatic site (brain, lymph node, and pleural effusion, respectively). Dot size indicates the percentage of cells expressing *NDUFA9* (pct.exp), and color intensity represents the average expression level (avg.exp). The malignant epithelial cells consistently exhibit high *NDUFA9* expression across all examined metastatic locations.

To investigate the expression of *NDUFA9* in LUAD across various metastatic sites, we analyzed publicly available single-cell RNA sequencing data from the GSE131907 dataset, with cell type annotations provided by the original study authors [43]. Analysis of the LUAD brain metastasis (mBrain) samples (Fig. 3A, B) revealed a distinct enrichment of *NDUFA9* transcripts within the malignant epithelial cell population. Next, examination of the LUAD lymph node samples (Fig. 3C, D) demonstrated elevated *NDUFA9* expression specifically in the malignant epithelial cells, notably within the context of metastatic involvement in the lymph node (mLN). Furthermore, assessment of the LUAD pleural effusion (PE) samples (Fig. 3E, F) also indicated a prominent expression of *NDUFA9* within the malignant epithelial cell compartment. These observations consistently highlight a robust expression of *NDUFA9* in the malignant epithelial cells across diverse metastatic niches of LUAD, including the brain, lymph nodes, and pleural fluid.

### NDUFA9 upregulation in NSCLC tissues of locally-treated patients and various NSCLC cell types

**Fig. 4:**
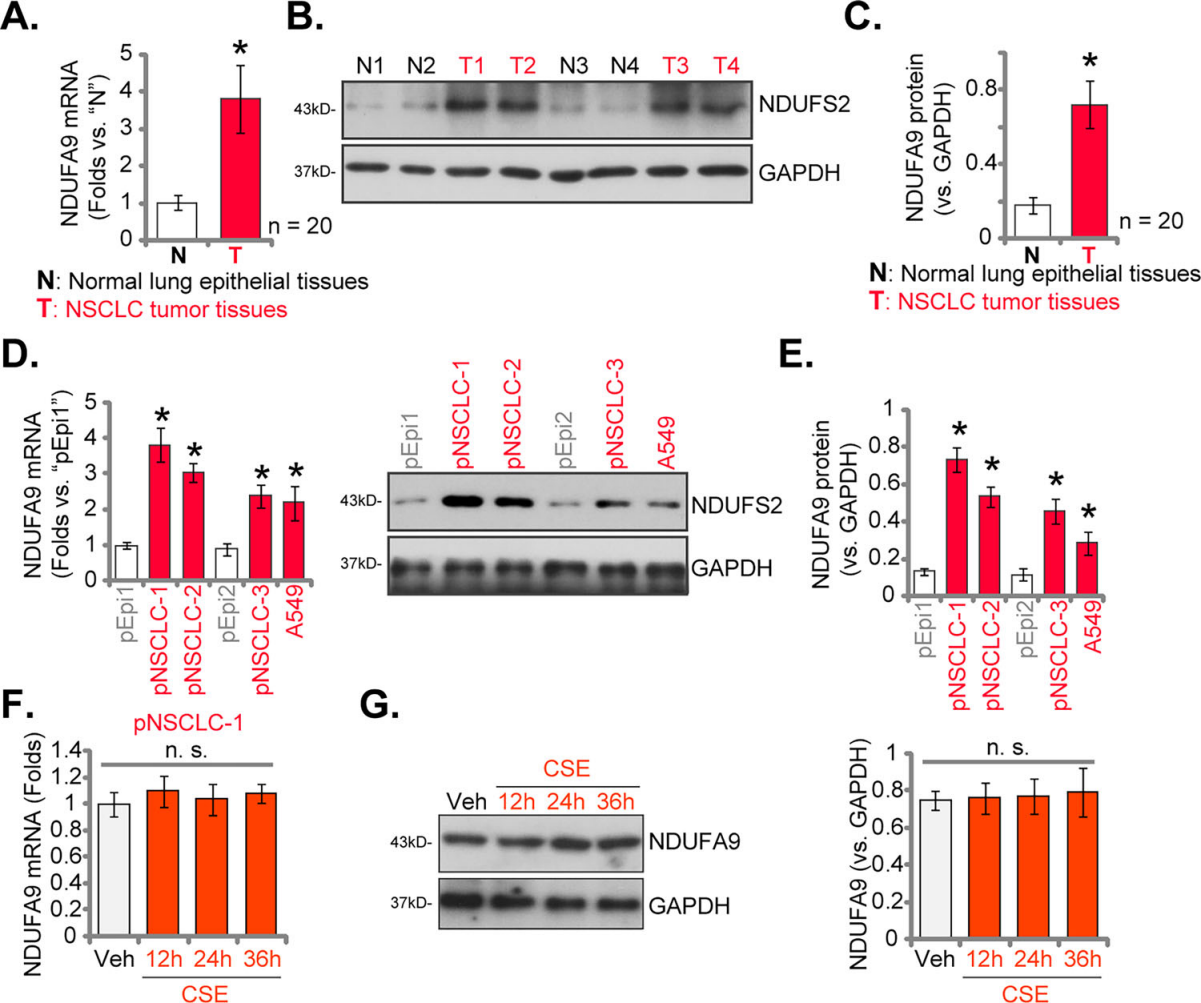
NDUFA9 upregulation in NSCLC tissues of locally-treated patients and various NSCLC cell types. *NDUFA9* mRNA levels were measured by qRT-PCR in NSCLC tumor tissues (T) compared to adjacent normal lung tissues (N) from a panel of twenty (*n* = 20) locally-treated NSCLC patients (**A**). Western blot analysis was performed to assess NDUFA9 protein expression in the listed NSCLC tumor tissues (T1-T4) compared to corresponding normal lung tissues (N1-N4) (**B**). A summary of blotting data for the 20 pairs of tissues is shown (**C**). *NDUFA9* mRNA expression levels in various NSCLC cell types (pNSCLC-1, pNSCLC-2, and pNSCLC-3 primary cells, and established A549 cell line) compared to non-cancerous lung epithelial cells (pEpi1 and pEpi2) were measured by qRT-PCR (**D**). Western blot analysis was performed to assess NDUFA9 protein expression in NSCLC cell types compared to non-cancerous lung epithelial cells (**E**). *NDUFA9* mRNA (**F**) and protein (**G**) levels in pNSCLC-1 cells treated with CSE (5%) or vehicle control (“Veh”) for the indicated time periods were tested. Data values are presented as the mean ± standard deviation (SD). Statistical significance was marked by ∗ *P* < 0.05 compared to the “N” tissues or “pEpi1” cells. “n.s.” denotes non-significant differences (*P* > 0.05).

Bioinformatic analyses, including studies from TCGA (see Fig. 1) and single-cell sequencing data (see Figs. 2 and 3), consistently revealed a significant upregulation of *NDUFA9* in NSCLC. To experimentally validate these findings, we next demonstrated that *NDUFA9* mRNA and protein expression is indeed significantly upregulated in both NSCLC tissues obtained from locally-treated patients and various NSCLC cell types. As depicted in Fig. 4A, *NDUFA9* mRNA levels were markedly elevated in NSCLC tumor tissues (T) compared to adjacent normal lung tissues (N) of a panel of twenty (*n* = 20) locally-treated patients. This transcriptional increase was further corroborated at the protein level, with Western blot analysis (Fig. 4B) confirming a substantial increase in NDUFA9 protein expression in NSCLC tumor tissues of four representative patients (Patient-1 to Patient-4). The summary of blotting data for all 20 pairs of tissues is shown in Fig. 4C, which illustrates that NDUFA9 protein expression in NSCLC tumor tissues is significantly upregulated when compared to the paired surrounding normal lung tissues. Furthermore, our investigations into various NSCLC cell types, including primary cancer cells derived from three patients, pNSCLC-1, pNSCLC-2 and pNSCLC-3 (provided by Dr. Sang [25, 32, 33]. as well as the A549 cell line, revealed a consistent and significant upregulation of *NDUFA9* mRNA (Fig. 4D) and protein (Fig. 4E) expression when compared to non-cancerous lung epithelial cells (pEpi1 and pEpi2, also provided by Dr. Sang [25, 32, 33]. These comprehensive findings from both patient samples and cell models firmly establish NDUFA9 as an aberrantly overexpressed gene/protein in NSCLC.

To determine if cigarette smoke exposure (CSE) affects NDUFA9 expression, pNSCLC-1 cells were treated with CSE (5%). The qRT-PCR analysis revealed that CSE exposure for 12, 24, or 36 hours did not significantly alter the mRNA levels of NDUFA9 compared to vehicle-treated controls (Fig. 4F). Consistent with these findings, Western blot analysis also showed no significant changes in NDUFA9 protein levels at the same time points (Fig. 4G).

### NDUFA9 silencing inhibits mitochondrial functions in NSCLC cells

**Fig. 5:**
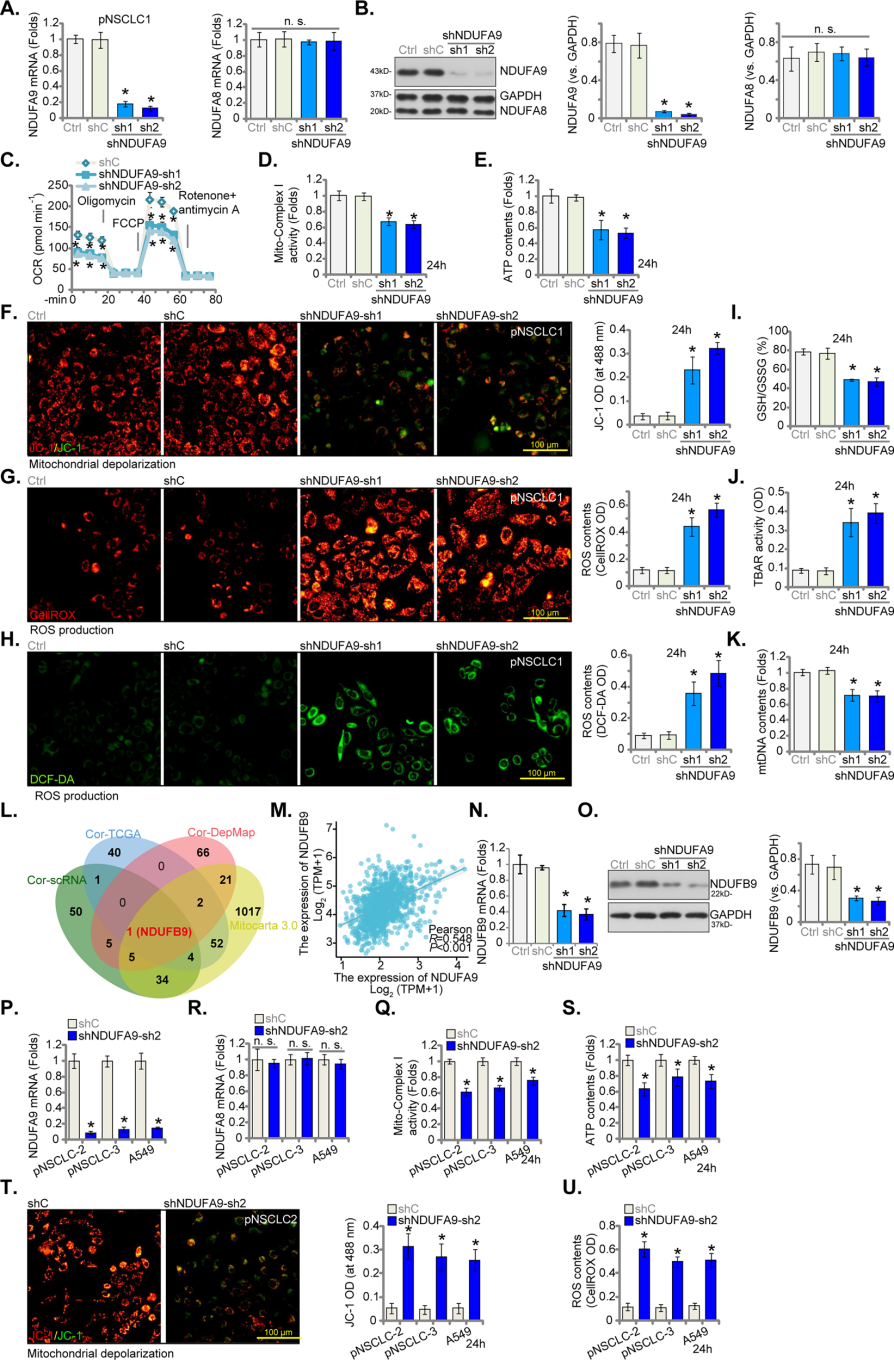
NDUFA9 silencing inhibits mitochondrial functions in NSCLC cells. Primary human NSCLC cells (pNSCLC1) were individually treated with targeted NDUFA9 shRNAs (shNDUFA9-sh1 and shNDUFA9-sh2, representing distinct sequences) or a control scramble shRNA (shC), and stable cells formed after selection, *NDUFA9* mRNA expression levels were measured by qRT-PCR (**A**) and protein expression levels by Western blot analysis (**B**) respectively, with NDUFA8 tested as a control gene (**A**, **B**). Cells were further cultivated for 24 h, oxygen consumption rate (OCR) was assessed by Seahorse XF analysis (**C**) and mitochondrial complex I activity (**D**) and cellular ATP production (**E**) were measured. Mitochondrial depolarization was assessed by JC-1 fluorescence (**F**). Reactive oxygen species (ROS) production was evaluated by CellROX (**G**) and DCF-DA staining (**H**) GSH/GSSG ratio (**I**), lipid peroxidation (TBARintensity, **J**) and mtDNA contents (**K**) were measured were quantified (**I**). Bioinformatic analysis, including the intersection of data from TCGA-NSCLC, DepMap proteomic data, single-cell sequencing studies, and the MitoCarta 3.0 database, identified *NDUFB9* as a common co-expressed mitochondrial gene of NDUFA9 (**L**) and a scatter plot illustrating the correlation between *NDUFA9* and *NDUFB9* expression within the TCGA-NSCLC cohort (**M**). *NDUFB9* mRNA (**N**) and protein (**O**) expression levels were measured by qRT-PCR and Western blot analysis, respectively, following NDUFA9 knockdown in pNSCLC1 cells. Two additional primary NSCLC cell types (pNSCLC-2 and pNSCLC-3) and the established A549 cell line were treated with shNDUFA9-sh2 or shC, with stable cells formed after selection. *NDUFA9* and *NDUFA8* expression (**P**, **Q**) were measured. Cells were further cultured for 24 h, the mitochondrial complex I activity (**R**) and cellular ATP production (**S**) were measured. Mitochondrial depolarization was assessed by JC-1 monomer accumulation (**T**) and ROS production was measured by increased CellROX intensity (**U**). “Ctrl” stands for the parental control cells. Data values are presented as the mean ± standard deviation (SD). Statistical significance was marked by ∗ *P* < 0.05 compared to the “shC” cells, while “n.s.” denotes non-significant differences (*P* > 0.05). These experiments were conducted five times (biological repeats), consistently producing similar results. Scale bar = 100 μm.

To elucidate the functional role of NDUFA9 in NSCLC cells, we employed a targeted gene silencing approach utilizing two distinct short hairpin RNAs (shRNAs), shNDUFA9-sh1 and shNDUFA9-sh2, in patient-derived primary human NSCLC cells (pNSCLC1). As depicted in Fig. 5A, qRT-PCR analysis revealed that both shNDUFA9-sh1 and shNDUFA9-sh2 significantly reduced *NDUFA9* mRNA expression compared to the shC control group, which was further corroborated by a substantial reduction in NDUFA9 protein levels (Fig. 5B). The expression of the control gene *NDUFA8* remained unaffected at both mRNA and protein levels (Fig. 5A, B). Subsequently, Seahorse XF analysis (Fig. 5C) indicated that the knockdown of NDUFA9 profoundly inhibited the oxygen consumption rate (OCR). Both basal respiration and maximal respiration (following FCCP injection) were markedly diminished in NDUFA9-silenced pNSCLC1 cells (Fig. 5C). Furthermore, mitochondrial complex I activity was compromised, exhibiting a significant decline in activity in both shNDUFA9-sh1- and shNDUFA9-sh2-treated pNSCLC1 cells (Fig. 5D). The cellular ATP production was also substantially (Fig. 5E). Beyond energetic impairment, NDUFA9 silencing also induced significant mitochondrial depolarization, as evidenced by a pronounced increase in the green JC-1 monomers, signifying a loss of mitochondrial membrane potential (Fig. 5F). The generation of reactive oxygen species (ROS) was significantly elevated, with CellROX and DCF-DA staining revealing a significant increase in fluorescence intensity in NDUFA9-knockdown pNSCLC1 cells (Fig. 5G, H). Moreover, GSH/GSSG ratio was decreased, lipid peroxidation (TBAR intensity) was increased, and mtDNA contents were decreased in NDUFA9-silenced pNSCLC1 cells, further suggesting compromised mitochondrial integrity (Fig. 5I, J, K).

To identify potential co-expressed mitochondrial genes of NDUFA9, a comprehensive multi-omic bioinformatics analysis was conducted. This analysis integrated top 100 co-expressed genes of *NDUFA9* from TCGA- NSCLC cohort (see Fig. 1), proteomic data of top 100 positively correlated proteins of NDUFA9 from DepMap, top 100 co-expressed genes of *NDUFA9* identified from single-cell sequencing studies (see Fig. 2), and a curated list of mitochondrial genes from the MitoCarta 3.0 database. The stringent intersection converged on a single, highly significant co-expressed molecule: NDUFB9 (Fig. 5L). The scatter plot illustrating the correlation between NDUFA9 and NDUFB9 expression within the TCGA-NSCLC cohort (Fig. 5M) revealed a strong positive correlation. Further experimental validation demonstrated that NDUFA9 silencing in pNSCLC1 cells resulted in a concomitant downregulation of both *NDUFB9* mRNA and protein expression (Fig. 5N, O), providing robust evidence for their interdependent expression and further supporting a role of NDUFA9 in mitochondrial function in NSCLC cells.

To ascertain the generalizability of these findings, the effects of NDUFA9 silencing were further investigated in two additional primary NSCLC cell types (pNSCLC-2 and pNSCLC-3) and the established A549 cell line. Consistent with observations in pNSCLC1 cells, shNDUFA9-sh2 effectively reduced *NDUFA9* mRNA expression in these NSCLC cells, without altering *NDUFA8* mRNA levels (Fig. 5P, Q). Importantly, NDUFA9 silencing consistently led to significant declines in mitochondrial complex I activity (Fig. 5R) and ATP production (Fig. 5S). Furthermore, NDUFA9 depletion induced pronounced mitochondrial depolarization (JC-1 monomers accumulation, Fig. 5T) and a significant increase in ROS production (CellROX intensity increasing, Fig. 5U) in these additional NSCLC cellular contexts. These consistent results across multiple NSCLC cell types underscore the critical role of NDUFA9 in maintaining mitochondrial integrity and function.

### NDUFA9 silencing inhibits malignant phenotypes in NSCLC cells

**Fig. 6:**
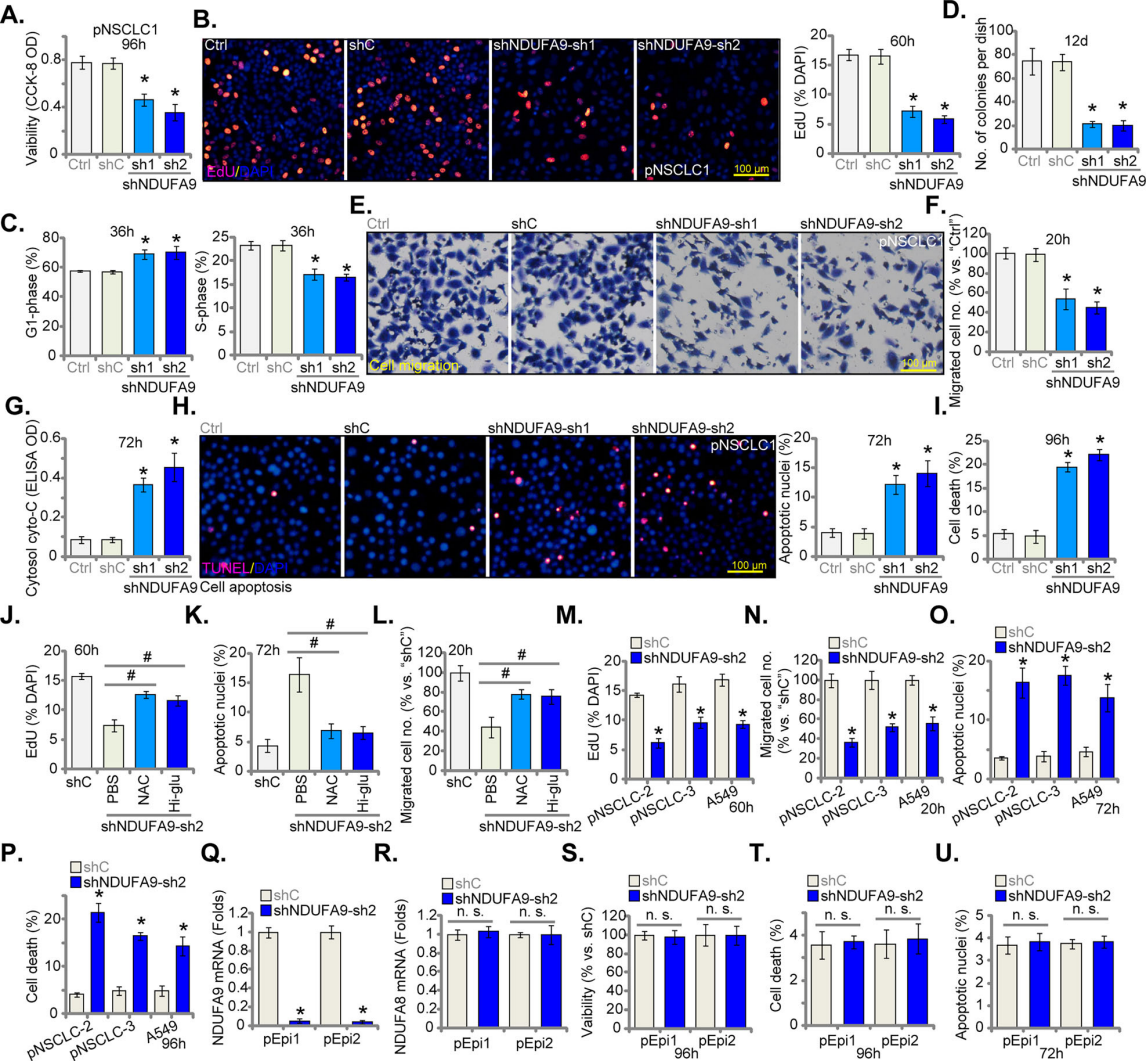
NDUFA9 silencing inhibits malignant phenotypes in NSCLC cells. Primary human NSCLC cells (pNSCLC1) were treated with targeted NDUFA9 shRNAs (shNDUFA9-sh1 and shNDUFA9-sh2) or a control scramble shRNA (shC), and stable cells formed after selection. Cells were cultured for designated time, with cell viability assessed by CCK-8 OD (**A**). Nuclear EdU incorporation was measured to assess DNA synthesis and proliferation (**B**). Cell cycle distribution was analyzed (**C**). Colony formation assays were performed to evaluate long-term proliferation and survival (**D**). Transwell migration (**E**, **F**) assays were conducted to assess migratory capability. The cytosol cytochrome C ELISA was utilized to quantify apoptosis (**G**) and TUNEL staining was performed to detect apoptotic nuclei (**H**). Cell death was quantified by trypan blue staining analysis (**I**). The effects of shNDUFA9-sh2 on pNSCLC1 cell proliferation (**J**) apoptosis (**K**) and migration (**L**) were assessed in the presence of the antioxidant N-acetylcysteine (NAC, 0.5 mM) or increased glucose concentration (15 mM). shNDUFA9-sh2 was also applied to two additional primary NSCLC cell types (pNSCLC-2 and pNSCLC-3) and the A549 cell line. Nuclear EdU incorporation was measured (**M**). Transwell migration assays were performed (**N**). TUNEL staining was performed to detect apoptosis (**O**) with cell death quantified by Trypan blue staining (**P**). Two distinct non-cancerous primary human lung epithelial cells, pEpi1 and pEpi2 (derived two patients) were treated with shNDUFA9-sh2 or shC as well. *NDUFA*9 and *NDUFA8* mRNA levels were measured (**Q**, **R**). Cells were cultured for designated time, overall cell viability (**S**) the extent of cell death (**T**) and the apoptotic rate (**U**) were assessed using the similar methods. “Ctrl” stands for the parental control cells. Data values are presented as the mean ± standard deviation (SD). Statistical significance was marked by ∗ *P* < 0.05 compared to the “shC” cells, while “n.s.” denotes non-significant differences (*P* > 0.05). These experiments were conducted five times (biological repeats), consistently producing similar results. Scale bar = 100 μm.

We next examined the impact of NDUFA9 silencing on various malignant cellular phenotypes. As illustrated in Fig. 6A, the viability (CCK-8 OD) of pNSCLC1 cells was significantly compromised following the stable knockdown of NDUFA9 using both shNDUFA9-sh1 and shNDUFA9-sh2 (see Fig. 5). Figure 6B also demonstrated a marked reduction in nuclear EdU incorporation, providing robust evidence for the inhibition of DNA synthesis and, consequently, cell proliferation. Further detailed analysis of cell cycle distribution (Fig. 6C) revealed that NDUFA9 silencing led to the accumulation of cells in the G1 population, concurrently with a significant decrease in the S-phase population, again suggesting the induction of proliferation arrest and cell cycle arrest. Moreover, NDUFA9 knockdown significantly abrogated the intrinsic ability of pNSCLC1 cells to form colonies, underscoring its pivotal role in sustaining autonomous growth and long-term survival (Fig. 6D). Moreover, Transwell migration assays (Fig. 6E, F) demonstrated that NDUFA9 silencing substantially diminished the number of migrated cells. Beyond its effects on viability, proliferation, and migration, NDUFA9 knockdown also induced moderate but significant apoptosis in pNSCLC1 cells. TUNEL staining (Fig. 6H) revealed a modest increase (10-15%) in apoptotic nuclei percentage within shNDUFA9-treated cells, indicative of programmed cell death. This observation was further corroborated by the trypan blue staining analysis (Fig. 6I), which consistently showed a significant increase (20-25%) in the percentage of death cells. Furthermore, as demonstrated in Fig. 6J–L, the deleterious effects of shNDUFA9-sh2 on pNSCLC1 cells, including suppression of proliferation (Fig. 6J), the induction of apoptosis (Fig. 6K) and inhibition of migration (Fig. 6L), were significantly ameliorated by the addition of the antioxidant N-acetylcysteine (NAC) or by increasing glucose concentration in the cell culture medium. These findings suggest that the observed malignant phenotype inhibition by NDUFA9 silencing is mediated, at least in part, through mitochondrial dysfunction-caused metabolic alterations and oxidative stress in NSCLC cells.

To further generalize these findings, the impact of shNDUFA9-sh2 on malignant phenotypes was also assessed in two additional primary NSCLC cell types (pNSCLC-2 and pNSCLC-3) and the A549 cell line. As shown in Fig. 6M, NDUFA9 silencing consistently reduced nuclear EdU incorporation in these cell types, confirming the broad inhibition of proliferation. Similarly, Transwell migration assays (Fig. 6N) demonstrated a significant reduction in cell migration across pNSCLC-2, pNSCLC-3, and A549 cells. TUNEL staining (Fig. 6O) and Trypan blue cell death staining (Fig. 6P) assays consistently revealed increased apoptosis and cell death in these additional NSCLC cell types upon NDUFA9 knockdown. These comprehensive findings unequivocally establish that NDUFA9 is indispensable for maintaining the aggressive malignant characteristics of NSCLC cells.

We next investigated the effects of NDUFA9 silencing in two distinct non-cancerous primary human lung epithelial cell lines, pEpi1 and pEpi2. As depicted in Fig. 6Q, shNDUFA9-sh2 effectively reduced *NDUFA9* mRNA expression in both pEpi1 and pEpi2 cells, without affecting NDUFA8 mRNA expression (Fig. 6R). However, in striking contrast to the observations in NSCLC cells, NDUFA9 silencing exerted no significant effect on the overall viability (Fig. 6S) or the extent of cell death (Fig. 6T) in pEpi1 and pEpi2 cells. Furthermore, the apoptotic rate (Fig. 6U) remained unaltered in these non-cancerous cells following NDUFA9 knockdown. These differential results strongly suggest that the inhibitory effects of NDUFA9 silencing on malignant phenotypes are highly specific to NSCLC cells and do not significantly compromise the health or function of normal lung epithelial cells.

### CRISPR/Cas9-mediated NDUFA9 knockout impairs mitochondrial function and malignant phenotypes in NSCLC cells

**Fig. 7:**
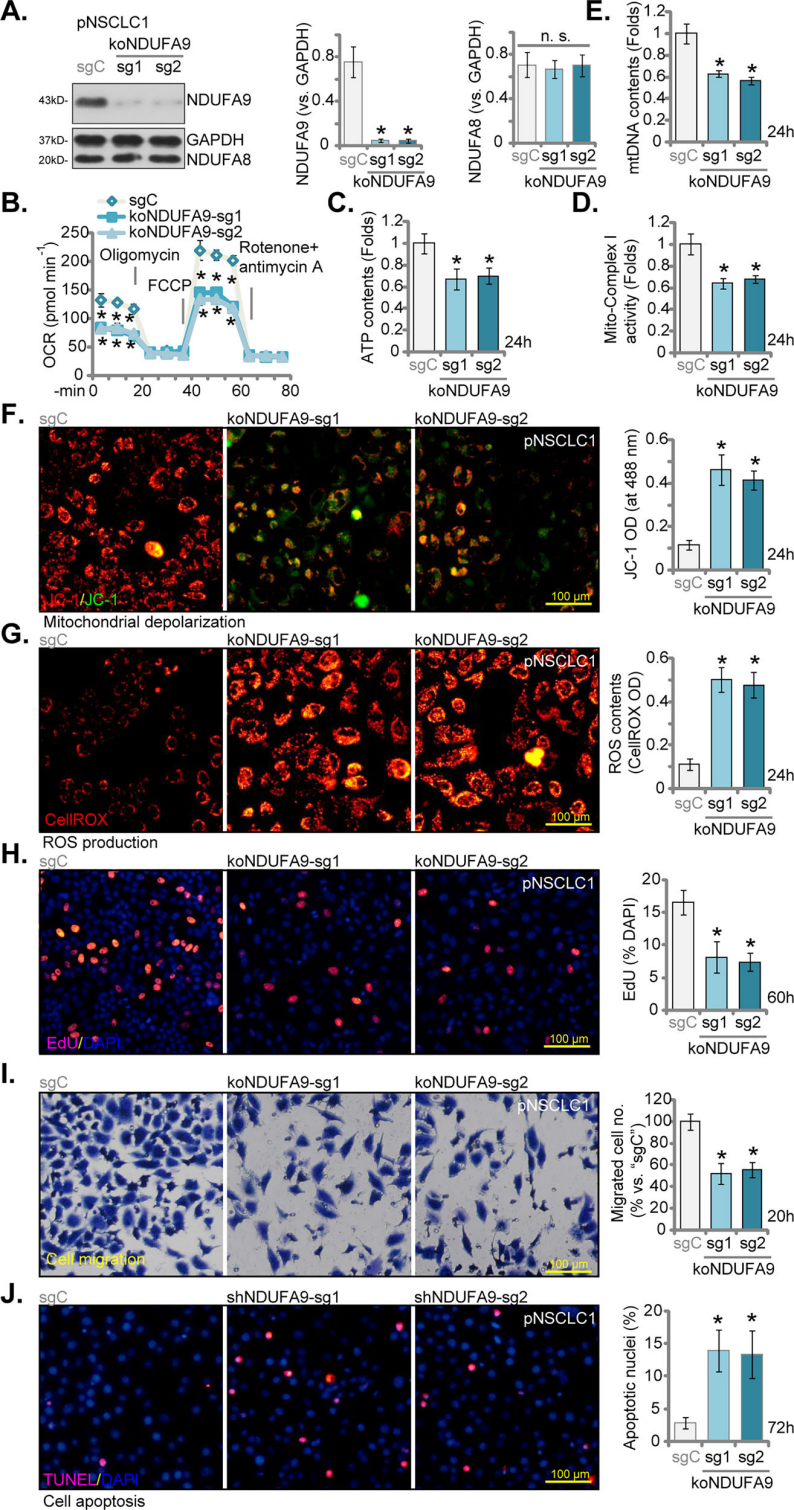
CRISPR/Cas9-mediated NDUFA9 knockout impairs mitochondrial function and malignant phenotypes in NSCLC cells. pNSCLC1 cells were engineered for stable NDUFA9 knockout (KO) using two distinct single guide RNAs (sgRNAs), koNDUFA9-sg1 and koNDUFA9-sg2, or a control scramble sgRNA (sgC). Western blot analysis was performed to confirm NDUFA9 protein expression, with NDUFA8 tested as a control (**A**). Cells were cultured for designated times and analyzed. Seahorse XF analysis was conducted to measure oxygen consumption rate (OCR) (**B**). Cellular ATP production (**C**) and mitochondrial complex I activity (**D**) were quantified. Mitochondrial DNA (mtDNA) contents were also measured (**E**). Mitochondrial depolarization was assessed by JC-1 monomers’ green fluorescence intensity (**F**) and reactive oxygen species (ROS) production was indicated by CellROX intensity (**G**). EdU staining assays were performed to assess cell proliferation (**H**). Transwell migration assays were conducted to evaluate migratory capability (**I**). TUNEL staining assay was performed to detect apoptotic nuclei ratio (**J**). Data values are presented as the mean ± standard deviation (SD). Statistical significance was marked by ∗ *P* < 0.05 compared to the “sgC” cells, while “n.s.” denotes non-significant differences (*P* > 0.05). These experiments were conducted five times (biological repeats), consistently producing similar results. Scale bar = 100 μm.

To further corroborate the role of NDUFA9 in NSCLC, we leveraged CRISPR/Cas9 gene editing technology to generate stable NDUFA9 knockout (KO) pNSCLC1 cells. This was achieved using two distinct single guide RNAs (sgRNAs), koNDUFA9-sg1 and koNDUFA9-sg2. As presented in Fig. 7A, Western blot analysis confirmed a significant and sustained reduction in NDUFA9 protein expression in koNDUFA9-sg1/2-treated pNSCLC1 cells, when compared to the sgC control cells, and NDUFA8 expression remained unaffected (Fig. 7A). The functional consequences of NDUFA9 knockout on mitochondrial bioenergetics were subsequently assessed. Seahorse XF analysis (Fig. 7B) revealed that NDUFA9 knockout profoundly inhibited the basal and maximum OCR, indicative of severe mitochondrial respiratory dysfunction. The cellular ATP production was significantly diminished in koNDUFA9 pNSCLC1 cells (Fig. 7C), and mitochondrial complex I activity experienced a substantial reduction (Fig. 7D). Furthermore, NDUFA9 knockout led to a significant decrease in mitochondrial DNA (mtDNA) content in pNSCLC1 cells (Fig. 7E), suggesting compromised mitochondrial biogenesis and stability. Beyond the observed bioenergetic impairment, NDUFA9 knockout induced significant mitochondrial depolarization, as evidenced by JC-1 green monomers accumulation (Fig. 7F), and markedly increased reactive oxygen species (ROS) production (CellROX intensity increasing, Fig. 7G). The impact of NDUFA9 knockout on the malignant phenotypes of pNSCLC1 cells was also evaluated. EdU staining assays (Fig. 7H) demonstrated a significant reduction in nuclear EdU incorporation in koNDUFA9 cells, providing evidence of impaired cell proliferation. Moreover, the migratory capability was severely compromised, as illustrated by Transwell migration (Fig. 7I) assays. TUNEL staining assay (Fig. 7J) revealed a moderate increase in apoptotic nuclei within koNDUFA9 cells, indicating that NDUFA9 knockout induced programmed cell death. Collectively, these results derived from CRISPR/Cas9-mediated NDUFA9 knockout experiments strongly corroborate the findings obtained from shRNA-mediated silencing, unequivocally establishing NDUFA9 as a crucial regulator of both mitochondrial function and malignant phenotypes in NSCLC cells.

### NDUFA9 overexpression enhances mitochondrial function and promotes malignant phenotypes in NSCLC cells

**Fig. 8:**
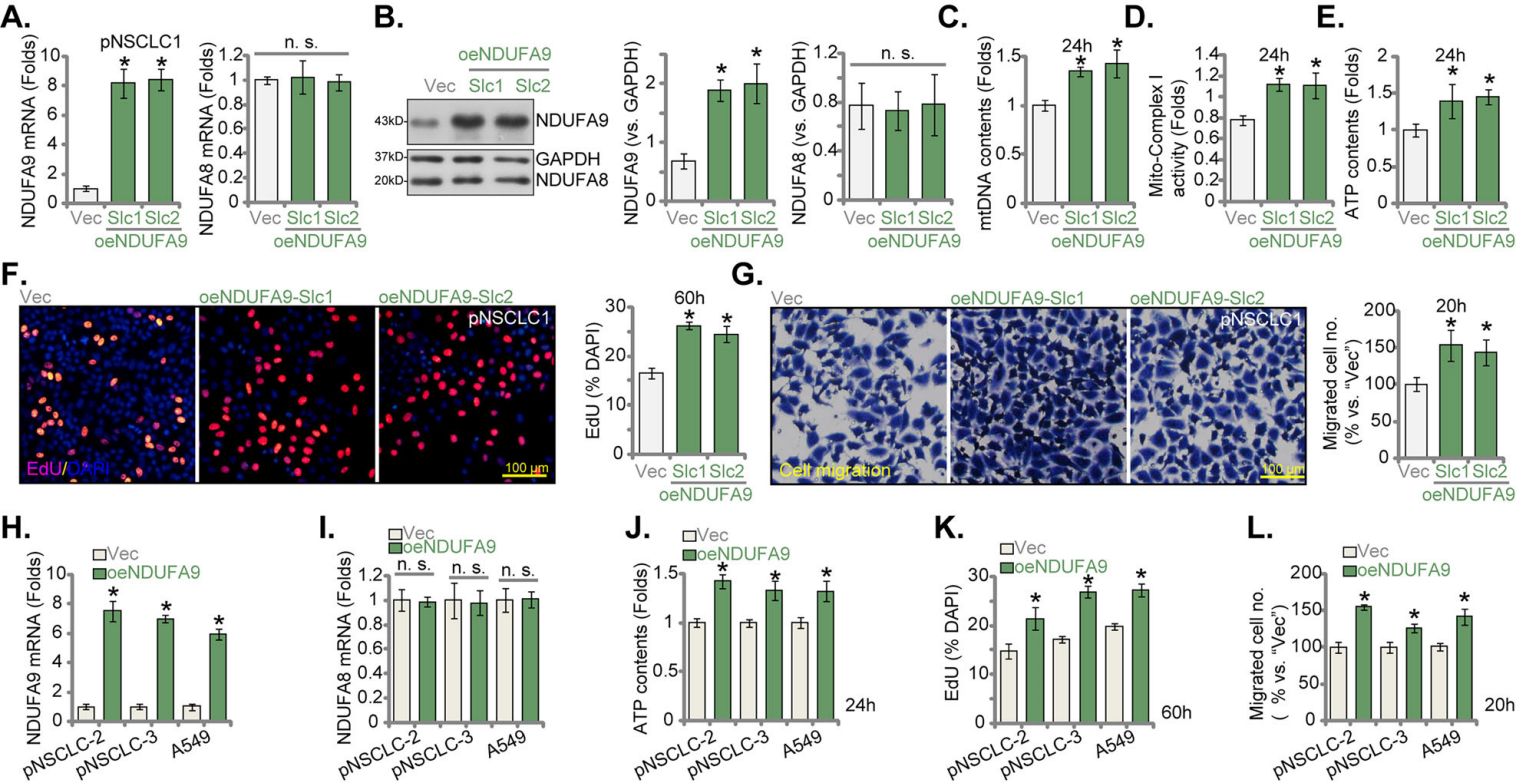
NDUFA9 overexpression enhances mitochondrial function and promotes malignant phenotypes in NSCLC cells. pNSCLC1 cells were transduced with a lentiviral NDUFA9-overexpressing construct, followed by selection to establish two stable cell lines, oeNDUFA9-Slc1 and oeNDUFA9-Slc2. Control cells were with an empty vector (Vec) control. *NDUFA9* mRNA (**A**) and protein (**B**) expression levels were measured by qRT-PCR and Western blot analysis, respectively, with NDUFA8 serving as a control gene (**A**, **B**). Cells were cultured for designated times and analyzed for mitochondrial DNA (mtDNA) contents (**C**) mitochondrial complex I activity (**D**) and cellular ATP production (**E**). EdU incorporation assays were performed to assess cell proliferation (**F**). Transwell migration assays were conducted to evaluate migratory capability (**G**). NDUFA9 was also overexpressed in two additional primary NSCLC cell types (pNSCLC-2 and pNSCLC-3) and the A549 cell line. *NDUFA9* and *NDUFA8* mRNA expression levels were measured by qRT-PCR (**H**, **I**). Cellular ATP production (**J**) and nuclear EdU incorporation (**K**) were quantified. Transwell migration assays were performed (**L**). Data values are presented as the mean ± standard deviation (SD). Statistical significance was marked by ∗ *P* < 0.05 compared to the “Vec” cells, while “n.s.” denotes non-significant differences (*P* > 0.05). These experiments were conducted five times (biological repeats), consistently producing similar results. Scale bar = 100 μm.

To complement our shRNA/KO studies and further elucidate the oncogenic role of NDUFA9 in NSCLC, we generated pNSCLC1 cells engineered for stable NDUFA9 overexpression. This was accomplished by transducing pNSCLC1 cells with a lentiviral NDUFA9-overexpressing construct, followed by rigorous selection to establish two stable cell selections, designated oeNDUFA9-Slc1 and oeNDUFA9-Slc2. Figure 8A confirmed a significant elevation in *NDUFA9* mRNA expression in both oeNDUFA9-Slc1 and oeNDUFA9-Slc2 pNSCLC1 cells when compared to the empty vector (Vec) control. This transcriptional upregulation was further corroborated by Western blot analysis (Fig. 8B). NDUFA8 expression remained unaffected (Fig. 8A, B). NDUFA9 overexpression consistently led to a significant increase in mitochondrial DNA (mtDNA) content (Fig. 8C), strongly suggesting enhanced mitochondrial biogenesis. Furthermore, mitochondrial complex I activity was significantly elevated in oeNDUFA9 pNSCLC1cells (Fig. 8D), and cellular ATP production was increased (Fig. 8E). The impact of NDUFA9 overexpression on the malignant phenotypes of pNSCLC1 cells was also evaluated. Results demonstrated a significant increase in nuclear EdU incorporation in oeNDUFA9 cells, providing clear evidence of augmented cell proliferation (Fig. 8D). Moreover, migratory ability was significantly promoted, as tested by Transwell assays (Fig. 8G). To further generalize these findings, the impact of NDUFA9 overexpression was also assessed in pNSCLC-2 and pNSCLC-3 primary cells and the A549 cell line. Figure 8H confirmed that *NDUFA9* mRNA expression was successfully increased in these NSCLC cells with the lentiviral NDUFA9-overexpressing construct (oeNDUFA9), and *NDUFA8* mRNA levels were unchanged (Fig. 8I). NDUFA9 overexpression consistently led to enhanced ATP production (Fig. 8J) and increased nuclear EdU incorporation (Fig. 8K). Similarly, the quantified Transwell migration assays (Fig. 8L) demonstrated a significant increase in cell migration across pNSCLC-2, pNSCLC-3, and A549 cells. These results collectively reinforce the broad applicability of NDUFA9’s role in promoting malignant phenotypes across diverse NSCLC cellular contexts.

### NDUFA9 positively modulates the Akt-mTOR cascade in NSCLC cells

**Fig. 9:**
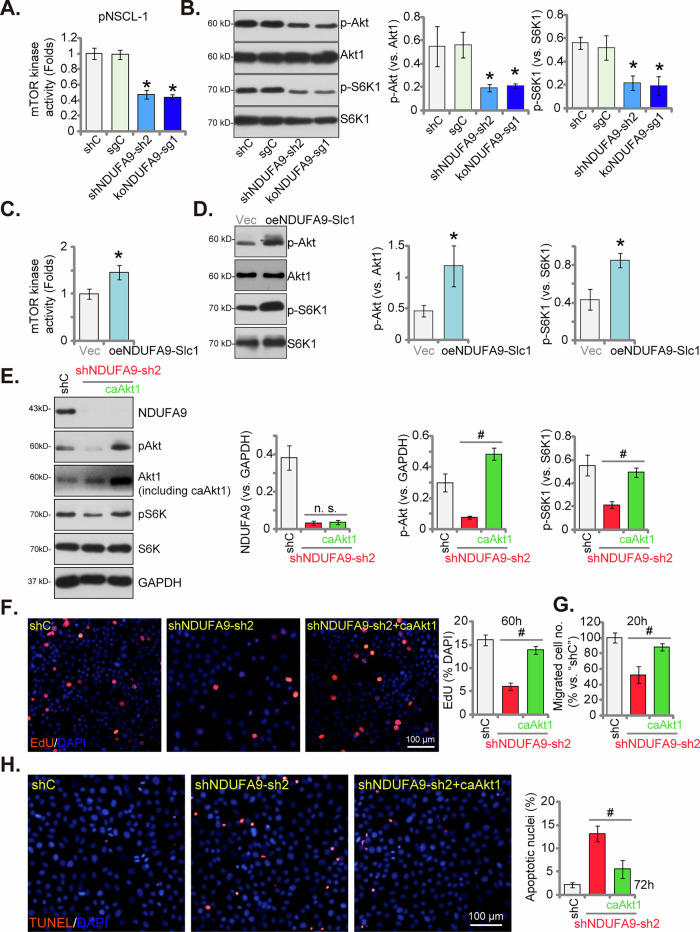
NDUFA9 positively modulates Akt-mTOR cascade in NSCLC cells. The primary human pNSCLC1 cells were treated with shNDUFA9-sh2, koNDUFA9-sg1, or shC and sgC controls. mTOR kinase activity was measured (**A**). Western blot analysis was performed to assess the phosphorylation levels of Akt (p-Akt) and S6K (p-S6K), as well as total Akt and S6K protein levels (**B**). pNSCLC1 cells were transduced with a lentiviral NDUFA9-overexpressing construct (oeNDUFA9-Slc1) or an empty vector (Vec) control. mTOR kinase activity was measured (**C**). Western blot analysis was performed to assess the phosphorylation levels of Akt (p-Akt) and S6K (p-S6K), as well as total Akt and S6K protein levels (**D**). A constitutively active Akt1 (caAkt1) was introduced into NDUFA9-silenced pNSCLC1 cells (with shNDUFA9-sh2). Western blot analysis was performed to test NDUFA9, Akt, p-Akt, S6K, and p-S6K protein levels (**E**). Cells were cultured for designated times and analyzed for various parameters, specifically, nuclear EdU incorporation for cell proliferation (**F**) Transwell assays for cell migration (**G**) and TUNEL staining for apoptosis (**H**) were assessed in shNDUFA9-sh2 cells with and without caAkt1. Data values are presented as the mean ± standard deviation (SD). Statistical significance was marked by ∗ *P* < 0.05 compared to the “shC”/“Vec” cells, while “n.s.” denotes non-significant differences (*P* > 0.05). # *P* < 0.05 (**E**–**H**). These experiments were conducted five times (biological repeats), consistently producing similar results. Scale bar = 100 μm.

Given that ATP serves as an essential phosphate donor for mTOR kinase activity [36–38]. which is crucial for the catalytic phosphorylation of its substrates, including Akt and S6K, and the established role of the Akt-mTOR pathway overactivation in the progression of NSCLC [44–46]. We aimed to investigate the potential influence of NDUFA9 on Akt-mTOR activation within NSCLC cells. As demonstrated in Fig. 9A, both shRNA-mediated silencing (using shNDUFA9-sh2, see Figs. 5 and 6) or CRISPR/Cas9-induced knockout (using koNDUFA9-sg1, see Fig. 7) of NDUFA9 in pNSCLC1 cells significantly inhibited mTOR kinase activity. This inhibition was further corroborated by Western blot analysis (Fig. 9B), which revealed a marked reduction in the phosphorylation levels of Akt (p-Akt, at Ser-473) and S6K (p-S6K, at Thr-389), key downstream targets of mTOR (Fig. 9B). Conversely, the overexpression of NDUFA9 in pNSCLC1 cells (oeNDUFA9-Slc1, see Fig. 8) resulted in a significant increase in mTOR kinase activity (Fig. 9C). This was accompanied by elevated phosphorylation of both Akt (p-Akt) and S6K (p-S6K) (Fig. 9D), further solidifying the positive regulatory role of NDUFA9 on the Akt-mTOR pathway in pNSCLC1 cells. To further investigate the functional interplay between NDUFA9 and Akt-mTOR signaling, we introduced a constitutively active Akt1 (caAkt1, S473D [41, 47]. into NDUFA9-silenced pNSCLC1 cells (shNDUFA9-sh2). Western blot analysis (Fig. 9E) confirmed that the introduction of caAkt1 effectively restored the phosphorylation of both Akt and S6K, despite the sustained knockdown of NDUFA9 (Fig. 9E). Importantly, this restoration of Akt-S6K phosphorylation significantly abrogated the anti-NSCLC cell activities induced by NDUFA9 shRNA (shNDUFA9-sh2). As depicted in Fig. 9F, the reduced nuclear EdU incorporation (indicative of inhibited proliferation) observed in shNDUFA9-sh2 cells was largely inhibited by caAkt1. Similarly, the inhibition of cell migration (Fig. 9G) and the induction of apoptosis (Fig. 9H) caused by shNDUFA9-sh2 were substantially ameliorated upon the introduction of caAkt1. These results strongly suggest that NDUFA9 exerts its pro-malignant effects in NSCLC cells, at least in part, by positively modulating the Akt-mTOR signaling pathway.

### YY1 is a key transcription factor regulating NDUFA9 expression in NSCLC cells

**Fig. 10:**
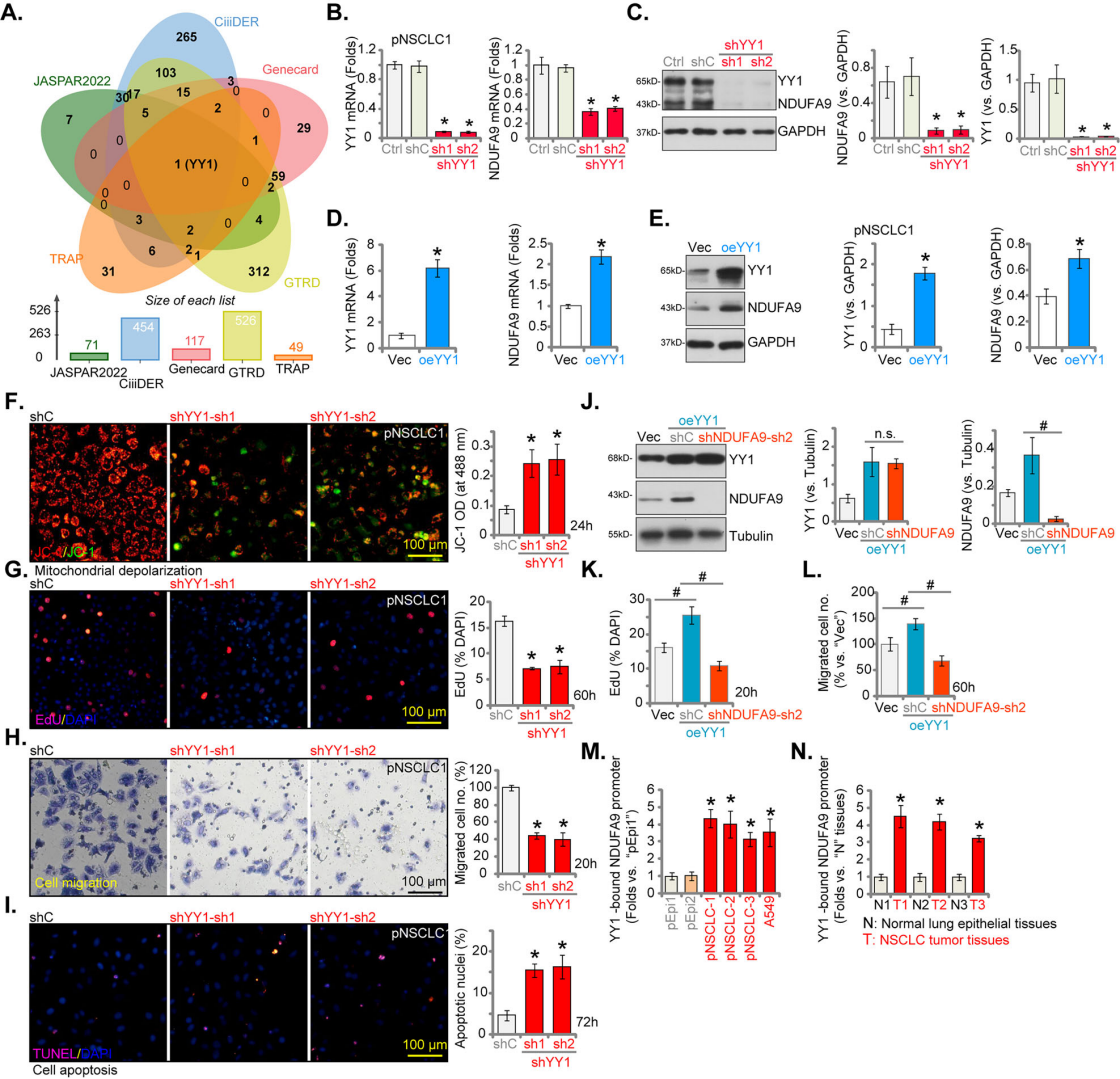
YY1 is a key transcription factor regulating NDUFA9 expression in NSCLC cells. A Venn diagram illustrating the intersection of predicted *NDUFA9* transcription factors from UCSC-JASPAR2022, GTRD, Genecard, CiiiDER, and TRAP databases, identifying YY1 as a common transcription factor (**A**). pNSCLC1 cells were treated with two different shRNAs targeting YY1 (sh1 and sh2) or a control scramble shRNA (shC). *YY1* and *NDUFA9* mRNA expression levels were measured by qRT-PCR (**B**). Western blot analysis was performed to assess YY1 and NDUFA9 protein levels (**C**). pNSCLC1 cells were transduced with a YY1-overexpressing construct (oeYY1) or an empty vector (Vec) control. *YY1* and *NDUFA9* mRNA expression levels were measured by qRT-PCR (**D**). Western blot analysis was performed to assess YY1 and NDUFA9 protein levels (**E**). pNSCLC1 cells with the two different shRNAs targeting YY1 (sh1 and sh2) or shC were cultured for designated times and analyzed. Mitochondrial depolarization was assessed by JC-1 monomers’ green fluorescence intensity (**F**) and EdU staining assays were performed to assess cell proliferation (**G**). Transwell migration (**H**) assays were conducted to evaluate migratory capability. TUNEL staining assay was performed to detect apoptotic nuclei ratio (**I**). pNSCLC1 cells with oeYY1 were further stably transduced with shNDUFA9-sh2 or shC, control cells were with empty vector (Vec). Western blot analysis was performed to assess YY1 and NDUFA9 protein levels (**J**). Cells were further cultured for designated times, cell proliferation (**K**) and migration (**L**) were tested using the same methods, with results quantified. Chromatin immunoprecipitation (ChIP) experiments were performed to assess YY1 binding to the predicted *NDUFA9* promoter region in various NSCLC cell types (pNSCLC-1, pNSCLC-2, pNSCLC-3, A549) compared to non-cancerous lung epithelial cells (pEpi1 and pEpi2) (**M**) and in human NSCLC tumor tissues (T) compared to adjacent normal lung tissues (**N**) from four patients (**N**). Data values are presented as the mean ± standard deviation (SD). Statistical significance was marked by ∗ *P* < 0.05 compared to the “shC”/“Vec” cells, or compared to “pEpi1” cells and “N” tissues. ^#^ *P* < 0.05 (**J**–**L**). “n.s.” denotes non-significant differences (*P* > 0.05). These experiments were conducted five times (biological repeats), consistently producing similar results.

Given the consistent and pronounced upregulation of both mRNA and protein levels of NDUFA9 observed across various NSCLC cell types and patient tissues, our investigation aimed to elucidate the specific transcriptional mechanism responsible for regulating its expression. To this end, a comprehensive bioinformatics prediction analysis was initially conducted utilizing five distinct and widely recognized databases: UCSC-JASPAR2022, GTRD, Genecard, CiiiDER, and TRAP. Adhering strictly to the operational guidelines of each platform, these databases were employed to predict potential transcriptional regulators of human *NDUFA9*. The rigorous intersection of these diverse predictions, visually represented in the Venn diagram in Fig. 10A, consistently converged upon a single common upstream transcription factor for NDUFA9: YY1. To experimentally validate the predicted regulatory role of YY1 on NDUFA9 expression, we precisely manipulated YY1 expression levels within pNSCLC1 primary cells. As depicted in Fig. 10B, C, two distinct shRNAs specifically targeting YY1 (shYY1-sh1 and shYY1-sh2) effectively achieved a significant reduction in *YY1* mRNA and protein expression. This targeted knockdown of YY1 subsequently resulted in a marked and consistent decrease in *NDUFA9* mRNA expression (Fig. 10B), accompanied by a corresponding and discernible reduction in NDUFA9 protein levels (Fig. 10C). Conversely, the forced overexpression of YY1 in pNSCLC1 cells (oeYY1) led to a significant elevation in *YY1* mRNA and protein expression (Fig. 10D, E), which in turn induced increased *NDUFA9* mRNA expression (Fig. 10D) and augmented NDUFA9 protein levels (Fig. 10E). These reciprocal findings demonstrate that YY1 positively regulates NDUFA9 expression in NSCLC cells.

To further investigate the role of the NDUFA9 transcriptional regulator, YY1, in NSCLC malignancy, we performed functional studies. Similar to the effects of NDUFA9 depletion, YY1 silencing induced significant mitochondrial depolarization, evidenced by the accumulation of JC-1 green monomers (Fig. 10F). YY1 knockdown also led to a significant inhibition of cell proliferation, measured by the decrease in EdU incorporation (Fig. 10G). Furthermore, Transwell assays demonstrated that YY1 silencing resulted in a substantial reduction in cell migration compared to the scrambled control (shC) (Fig. 10H). YY1 shRNA also caused a marked induction of apoptosis (Fig. 10I), quantified by the increase in TUNEL-positive nuclei. We next conducted rescue experiments to definitively place NDUFA9 downstream of YY1 signaling. Compared to vector controls (Vec), YY1 overexpression (Fig. 10J) significantly promoted cell proliferation (Fig. 10K) and enhanced cell migration (Fig. 10L), confirming the pro-malignant role of YY1 in pNSCLC-1 cells. Crucially, the simultaneous silencing of NDUFA9 using shNDUFA9-sh2 (oeYY1 + shNDUFA9-sh2, Fig. 10J) resulted in the complete reversal of the YY1 overexpression-induced increase in both cell proliferation (EdU assays) and cell migration (Transwell assays) (results quantified Fig. 10K, L). Thus, NDUFA9 is the essential downstream effector through which the transcription factor YY1 executes its pro-oncogenic activity to support NSCLC malignancy.

The results of the ChIP assay (Fig. 10M) provided compelling evidence of significantly increased binding between YY1 protein and the proposed *NDUFA9* promoter region. This enhanced binding affinity was consistently observed across multiple NSCLC cell types (pNSCLC-1, pNSCLC-2, pNSCLC-3 primary cells, and established A549 cell line) when compared to non-cancerous lung epithelial cells (pEpi1 and pEpi2). Furthermore, this elevated YY1 binding to the *NDUFA9* promoter was also significantly detected in various human NSCLC tumor tissues (Fig. 10N) in contrast to adjacent normal lung tissues. Thus, YY1 directly binds to and transcriptionally regulates the *NDUFA9* promoter, thereby contributing to its aberrant elevated expression in NSCLC.

### NDUFA9 silencing inhibits NSCLC xenograft tumor growth in nude mice

**Fig. 11:**
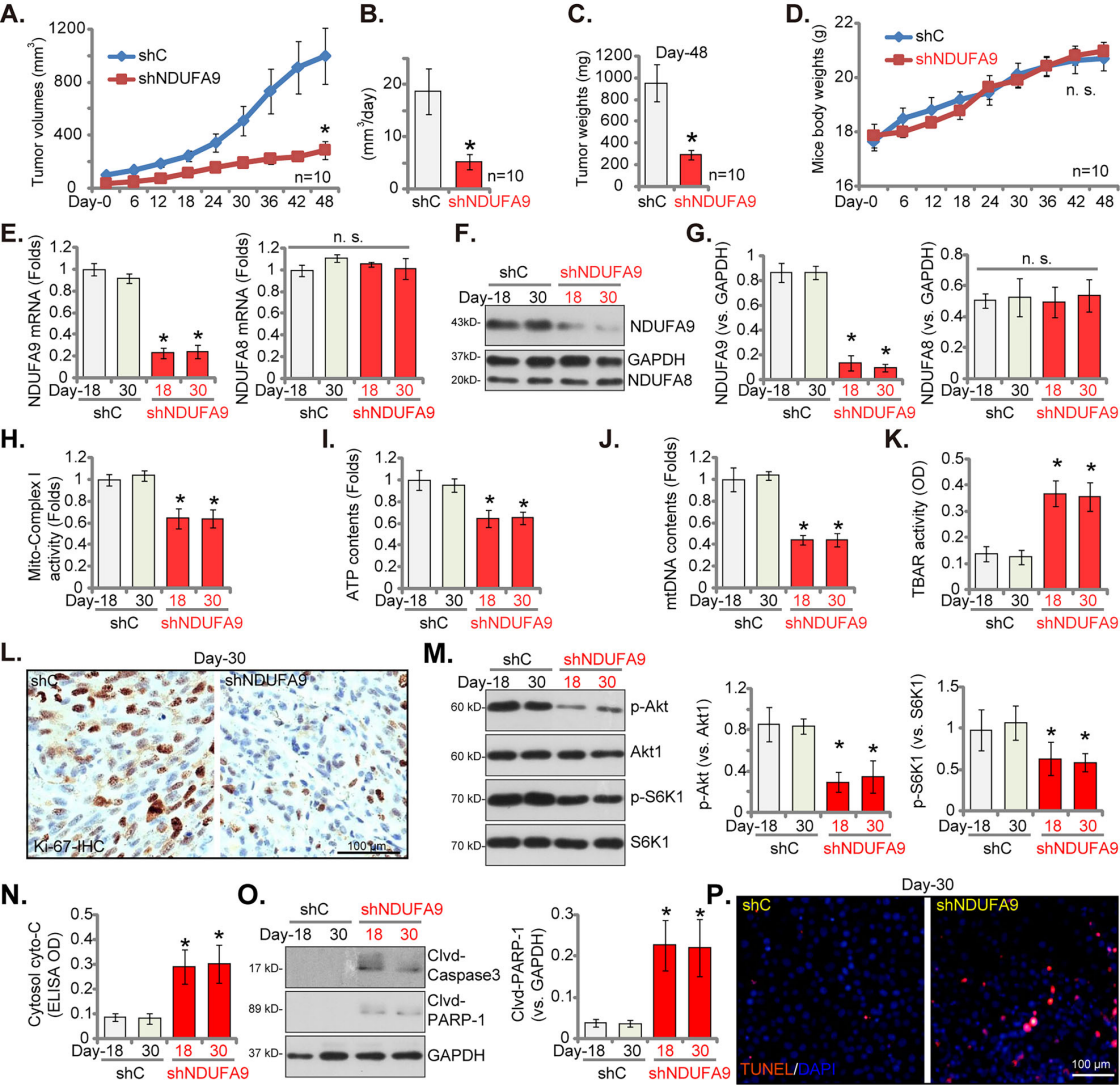
NDUFA9 silencing inhibits NSCLC xenograft tumor growth in nude mice. pNSCLC1 cells stably expressing either shNDUFA9-sh2 or a scramble control (shC) were subcutaneously injected into nude mice to establish xenograft tumors. Tumor growth was monitored starting three weeks post-injection (Day-0). Tumor volumes (**A**) tumor growth rate (in mm^3^ per day, **B**) and tumor weights at Day-48 (**C**) were measured. Mouse body weights were also monitored (**D**). Xenograft tumors were isolated from each group at experimental Day-18 and Day-30. *NDUFA9* mRNA (**E**) and protein (**F**, **G**) expression levels were measured by qRT-PCR and Western blot analysis, respectively, with NDUFA8 serving as a control gene (**E**–**G**). Mitochondrial complex I activity (**H**) ATP contents (**I**) and mitochondrial DNA (mtDNA) contents (**J**) were quantified, and TBAR activity was also measured (**K**). Tumor proliferation was assessed by Ki-67 immunohistochemical staining in xenograft slides (**L**). Western blot analysis of tumor lysates was performed to assess the phosphorylation levels of Akt (p-Akt) and S6K (p-S6K), as well as total Akt and S6K protein levels (**M**). Apoptosis was assessed by measuring cytoplasmic cytochrome c levels (**N**) cleavages of Caspase-3 and PARP-1 (**O**) in xenograft tissues, and TUNEL fluorescence staining in tumor slides (**P**). All data are presented as the mean ± standard deviation (SD). An asterisk (*) denotes statistical significance (*P* < 0.05) when compared to shC-treated xenografts, while “n.s.” indicates a non-significant difference (*P* > 0.05). *P*anels **A**–**D** include data from 10 mice per group. For **E**–**P**, analyses were conducted on five randomly selected tissue samples per xenograft (*n* = 5). The scale bar is 100 μm.

At last, we established a xenograft tumor model utilizing nude mice. pNSCLC1 cells stably expressing either shNDUFA9-sh2 or a scramble control (shC) were subcutaneously injected. Tumor growth was meticulously monitored commencing three weeks post-injection, designated as Day-0. As depicted in Fig. 11A, shNDUFA9-sh2-expressing pNSCLC1 xenograft tumors exhibited significantly attenuated growth rates compared to the shC control group, evidenced by a marked reduction in tumor volumes over the experimental duration (Fig. 11A, B). This substantial inhibition of tumor progression was further corroborated by significantly decreased tumor weights at the xenograft study’s conclusion (Day-48) (Fig. 11C). Importantly, the body weights of the mice remained unaffected throughout the experiment (Fig. 11D). To delineate the molecular mechanisms underpinning the observed in vivo tumor growth inhibition, xenograft tumors were carefully isolated from each experimental group at Day-18 and Day-30. As presented in Fig. 11E, *NDUFA9* mRNA expression was significantly diminished in shNDUFA9-sh2-expressing pNSCLC1 xenografts compared to shC controls, while *NDUFA8* mRNA levels remained unaltered (Fig. 11E). This reduction in NDUFA9 expression was also definitively confirmed at the protein level via Western blot analysis (Fig. 11F, G).

Consistent with our in vitro investigations, NDUFA9 silencing within the xenograft tumors led to a profound inhibition of mitochondrial function. The mitochondrial complex I activity (Fig. 11H), ATP contents (Fig. 11I), and mtDNA contents (Fig. 11J) were all significantly reduced in shNDUFA9-sh2 xenografts. ROS production, as quantified by TBAR activity (Fig. 11K), was significantly elevated. Moreover, NDUFA9 silencing significantly suppressed tumor cell proliferation, as evidenced by reduced Ki-67 immunohistochemical staining (Fig. 11L) within shNDUFA9-sh2 xenografts. The Akt-mTOR signaling pathway, which we had previously identified as being modulated by NDUFA9 in cultured pNSCLC1 cells, also demonstrated inhibition in vivo. Western blot analysis of tumor lysates (Fig. 11M) revealed diminished phosphorylation of Akt (p-Akt) and S6K (p-S6K) in shNDUFA9-sh2 xenografts, indicating reduced Akt-mTOR activation. NDUFA9 silencing also induced apoptosis within the xenograft tumors. This was demonstrated by increased cytoplasmic cytochrome c levels (Fig. 11N), elevated cleavage of Caspase-3 and PARP-1 (Fig. 11O), and an increase in TUNEL-positive apoptotic nuclei (Fig. 11P). Thus, NDUFA9 plays a crucial role in promoting NSCLC tumor growth by critically regulating mitochondrial function, cell proliferation, Akt-mTOR signaling, and apoptosis.

## Discussion

Despite advancements in treatment, the prognosis for NSCLC patients remains challenging, highlighting the critical importance of identifying and validating new molecular targets to improve patient outcomes [48, 49]. Several discrete mitochondrial proteins have been elucidated as pivotal orchestrators in NSCLC’s oncogenic trajectory. ADCK2 (aarF domain-containing kinase 2), a mitochondrial enzyme indispensable for coenzyme Q biogenesis and fatty acid catabolism, is consistently upregulated within NSCLC, with this elevated expression correlating with poor patient prognosis [23]. The inner mitochondrial membrane protease YME1L (YME1 Like 1 ATPase) is unequivocally upregulated in NSCLC [18]. Its genetic ablation abrogated NSCLC cell proliferation and migration, concomitantly inducing apoptosis and mitochondrial dysfunctionality, and attenuating xenograft tumorigenesis in vivo [22]. POLRMT (RNA polymerase mitochondrial), an enzyme transcendently vital for mtDNA transcription and thus integral to both mitochondrial protein synthesis and cellular bioenergetic output, is overexpressed in NSCLC and is demonstrably critical for NSCLC cell progression, both in vitro and in vivo [24]. MTCH2 (mitochondrial carrier homolog 2) is also overexpressed in NSCLC, correlating with an unfavorable patient prognosis; its functional perturbation profoundly inhibited NSCLC cell proliferation, migration, and invasion, while disrupting mitochondrial functionality [25]. Recently, Zha et al., identified TIMM23 (translocase of inner mitochondrial membrane 23) as a novel pro-tumorigenic determinant in NSCLC, demonstrating that its overexpression actively promoted tumor growth and progression by sustaining mitochondrial hyperfunction [20].

Our comprehensive findings provide compelling evidence that NDUFA9 represents a significant and promising therapeutic target in NSCLC. The consistent upregulation of NDUFA9 expression, observed across diverse bioinformatic datasets, including TCGA and single-cell RNA sequencing analyses, and subsequently validated in both NSCLC patient tissues and various cell types, strongly underscores its profound clinical relevance. Furthermore, the robust correlation of NDUFA9 expression with unfavorable clinicopathological indicators, such as advanced pathological T stage, male gender, smoking history, and diminished overall survival, unequivocally reinforces its prognostic significance. In primary or established human NSCLC cells, NDUFA9 shRNA or KO significantly suppressed key malignant phenotypes, including cell proliferation, migration, and, while inducing modest apoptosis in NSCLC cells. Conversely, NDUFA9 overexpression in NSCLC cells promoted malignant cellular phenotypes. The compelling in vivo xenograft studies, which unequivocally demonstrated that NDUFA9 silencing significantly suppressed pNSCLC1 xenograft growth, further solidify its potential as a viable and impactful therapeutic target for NSCLC intervention.

Interestingly, while clinical data demonstrate a strong correlation between smoking status and elevated *NDUFA9* expression, our in vitro models revealed that acute exposure to CSE did not directly upregulate NDUFA9 expression in primary human NSCLC cells. This suggests that NDUFA9 overexpression is not a transient transcriptional response to smoke toxins. Rather, it appears to be a stable, adaptive alteration acquired during tumor evolution. We postulate that the chronic oxidative and metabolic stress characteristic of the smoker’s lung microenvironment imposes a rigorous selective pressure, favoring the survival and expansion of malignant clones with enhanced mitochondrial Complex I stability via NDUFA9 upregulation, capable of sustaining mitochondrial integrity and hyperfunction. By reinforcing mitochondrial activity and mitigating ROS-induced cytotoxicity, high NDUFA9 expression confers the metabolic plasticity and bioenergetic robustness required to drive oncogenic transformation and progression in the face of chronic environmental insult.

Using TALEN-mediated gene knockout in HEK293T cells, Stroud et al., demonstrate that NDUFA9 is crucial for the assembly and activity of mitochondrial complex I, specifically by stabilizing the connection between mitochondrial membrane and matrix arms [50]. Through homozygosity mapping and candidate gene analysis in a consanguineous pedigree, a novel homozygous missense mutation in the *NDUFA9* gene was identified as the underlying cause of neonatally fatal Leigh syndrome with complex I deficiency, which was further confirmed by functional complementation studies in patient fibroblasts [29]. Utilizing exome sequencing and fibroblast analysis in two patients with varying clinical severity, Baertling et al., elucidated the role of *NDUFA9* in complex I biogenesis, demonstrating that distinct homozygous missense variants lead to complex I deficiency, correlating with the observed phenotypic spectrum of *NDUFA9*-related mitochondrial disease [27]. A recent study by Liu et al., elucidates a novel regulatory mechanism wherein NDUFA9 enhanced mitochondrial function, thereby promoting the browning of white adipocytes [26]. The NDUFA9 subunit possesses a tightly bound, non-catalytic NADPH molecule that engages with Arginine-178 of the NDUFS7 subunit [28].

Our evidence supports the notion that NDUFA9 is indispensable for maintaining mitochondrial hyperfunction, a hallmark characteristic of NSCLC cells. Both shRNA-mediated gene silencing and CRISPR/Cas9-induced genetic knockout of NDUFA9 profoundly compromised mitochondrial function. This impairment was consistently evidenced by a reduced OCR, diminished mitochondrial complex I activity, decreased ATP production, reduced mtDNA contents, mitochondrial depolarization, and an augmented generation of ROS. Conversely, NDUFA9 overexpression consistently enhanced these critical mitochondrial functions. Mitochondrial dysfunction was also detected in NDUFA9-silenced pNSCLC1 xenograft tissues. These findings collectively suggest that NDUFA9 plays a crucial role in supporting the elevated metabolic demands characteristic of rapidly proliferating cancer cells, thereby contributing significantly to their sustained growth and survival. Indeed, NDUFA9 silencing-induced detrimental effects in primary NSCLC cells were substantially ameliorated by the administration of antioxidants or by increasing glucose availability.

The activation of the Akt-mTOR pathway emerges as a critical and functionally important downstream effector of NDUFA9, given its well-established role as a vital signaling cascade driving NSCLC progression [44, 51]. Mitochondrial proteins are critically involved in the activation of the Akt-mTOR signaling pathway. For instance, experimental suppression of ADCK2 significantly inhibited NSCLC cell malignant phenotypes, disrupted mitochondrial functions, and notably attenuated Akt-mTOR signaling [23]. Similarly, NDUFS8, a key subunit of mitochondrial complex I, is overexpressed in NSCLC and promotes tumor growth, proliferation, and radio-resistance by enhancing mitochondrial function and positively regulating Akt-mTOR signaling [21]. Our data unequivocally demonstrate that NDUFA9 positively regulates this pathway: its depletion consistently inhibited, and its overexpression enhanced, mTOR kinase activity and the phosphorylation of both Akt and S6K in primary NSCLC cells. Crucially, the observation that introducing a caAkt1 restored Akt-S6K phosphorylation and abrogated the anti-NSCLC effects induced by NDUFA9 knockdown further substantiated the direct and functional interplay between NDUFA9 and Akt-mTOR signaling in NSCLC cells. This compellingly suggests that NDUFA9 promotes malignant phenotypes, at least in part, by positively modulating the Akt-mTOR axis.

YY1 is overexpressed in lung cancer and promotes tumor growth and invasion [52]. Computational analysis of RNA and protein expression datasets also suggests that high YY1 expression and low RKIP expression are negatively correlated in lung cancer, serving as potential diagnostic and prognostic biomarkers [53]. RPTOR (the regulatory-associated protein of mTOR) is upregulated in brain metastasis of NSCLC and enhances cell invasiveness by activating through direct binding of YY1 to the SPHK2 promoter [54]. The lncRNA PLIC11 promoted NSCLC cell proliferation, migration, and metastasis by interacting with YY1 and transcriptionally activating PIWIL4 expression [55]. YY1 acts as an upstream regulator of miR-1260b, which is upregulated in NSCLC and promotes cell proliferation and survival by suppressing SOCS6 and activating the KIT signaling pathway [56]. YY1 cooperates with YAP1 to cause resistance to the cancer drug osimertinib in NSCLC cells; they achieve this by suppressing the gene DUSP1 [57]. The hepatotoxic component Diosbulbin B (DIOB) demonstrates potent anti-NSCLC activity by directly binding to and inhibiting the oncogene Yin Yang 1 (YY1), which subsequently triggers the tumor suppressor P53 [58].

Our study identifies YY1-mediated transcriptional control as a primary and critical mechanism underlying NDUFA9 upregulation in NSCLC. Manipulating YY1 expression directly and significantly impacted NDUFA9 levels in primary NSCLC cells. Most importantly, ChIP experiments provided direct and compelling evidence of significantly increased binding between the YY1 protein and the NDUFA9 promoter region in both NSCLC cells and patient tissues. This direct transcriptional regulation by YY1 likely contributes substantially to the observed aberrant overexpression of NDUFA9 in NSCLC, thereby providing a distinct and promising upstream target for therapeutic intervention.

## Supplementary information

Original data

## Data Availability

All data generated or analyzed in this study are contained within the manuscript and its accompanying supplementary materials.
