# Supplementary figures and images for: YY1-mediated NDUFA9 upregulation promotes NSCLC cell growth through mitochondrial and Akt-mTOR pathway modulation
Source: Cell Death Dis. 2026 Apr 21;17(1):526. doi: 10.1038/s41419-026-08562-y (PMC13230915; doi:10.1038/s41419-026-08562-y)

Figure S1. Uncropped blotting images.

Figure 4.

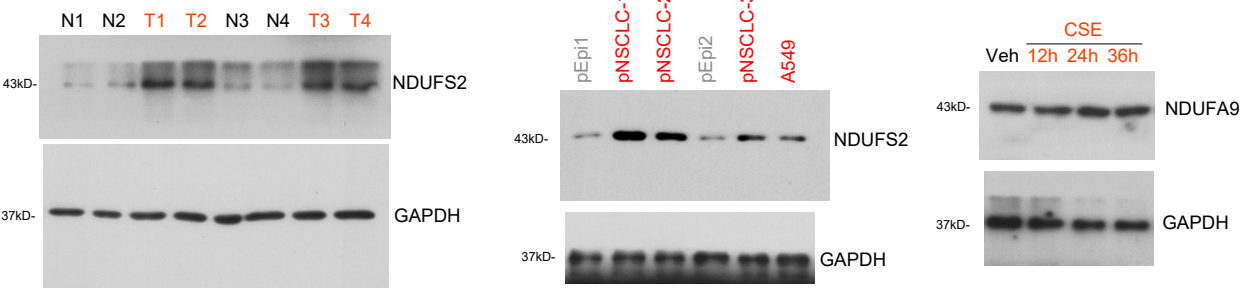

Figure 5.

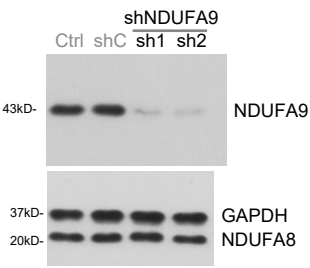

Figure 7.

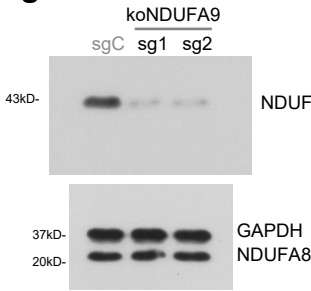

Figure 8.

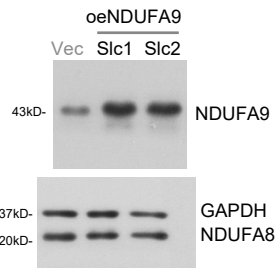

Figure 9.

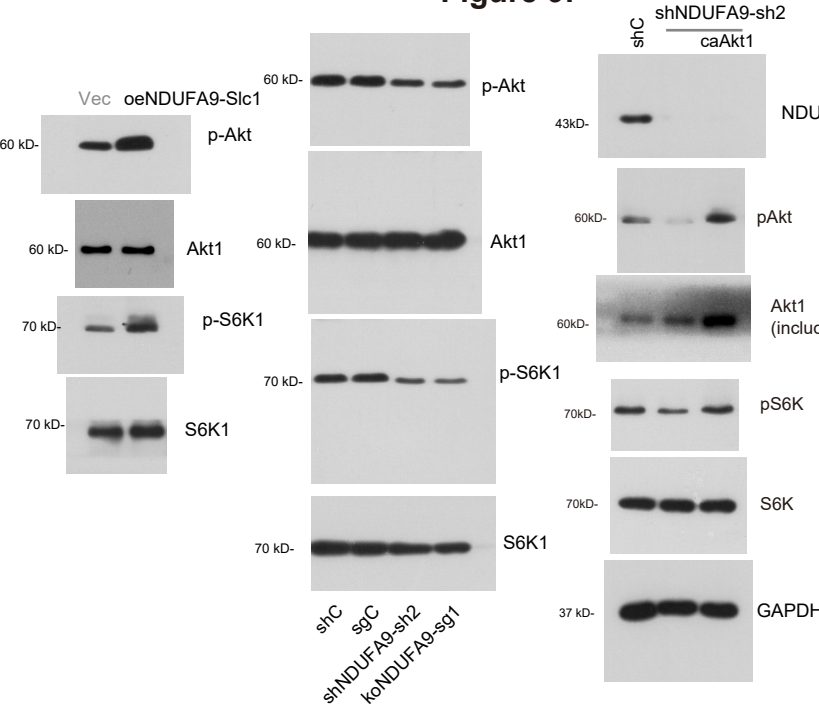

Figure 10.

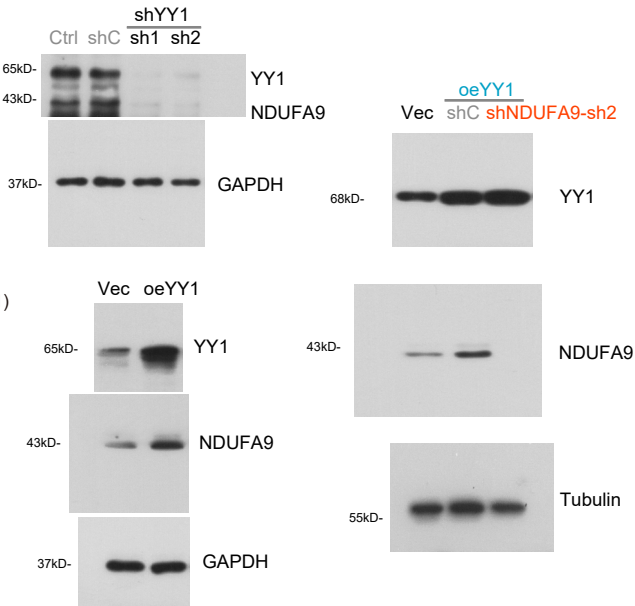

Figure 11.

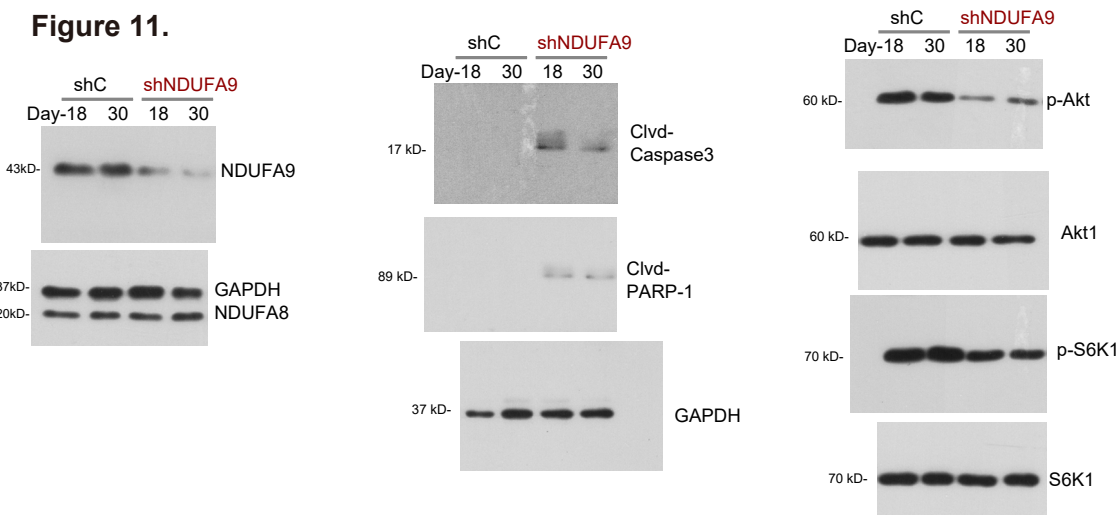

Supplement: Supplementary file 1 — Original data [file 41419_2026_8562_MOESM1_ESM.pdf]
